# Modeling Lewy body disease with *SNCA* triplication iPSC-derived cortical organoids and identifying therapeutic drugs

**DOI:** 10.1126/sciadv.adk3700

**Published:** 2024-09-11

**Authors:** Yunjung Jin, Fuyao Li, Zonghua Li, Tadafumi C. Ikezu, Justin O’Leary, Manikandan Selvaraj, Yiyang Zhu, Yuka A. Martens, Shunsuke Koga, Hannah Santhakumar, Yonghe Li, Wenyan Lu, Yang You, Kiara Lolo, Michael DeTure, Alexandra I. Beasley, Mary D. Davis, Pamela J. McLean, Owen A. Ross, Takahisa Kanekiyo, Tsuneya Ikezu, Thomas Caulfield, Jonathan Carr, Zbigniew K. Wszolek, Guojun Bu, Dennis W. Dickson, Na Zhao

**Affiliations:** ^1^ Department of Neuroscience, Mayo Clinic, Jacksonville, FL 32224, USA.; ^2^ Department of Clinical Trials and Biostatistics, Mayo Clinic, Jacksonville, FL 32224, USA.; ^3^ Tygerberg Hospital and University of Stellenbosch, Tygerberg 7505, South Africa.; ^4^ Department of Neurology, Mayo Clinic, Jacksonville, FL 32224, USA.

## Abstract

Aggregated α-synuclein (α-SYN) proteins, encoded by the *SNCA* gene, are hallmarks of Lewy body disease (LBD), affecting multiple brain regions. However, the specific mechanisms underlying α-SYN pathology in cortical neurons, crucial for LBD-associated dementia, remain unclear. Here, we recapitulated α-SYN pathologies in human induced pluripotent stem cells (iPSCs)–derived cortical organoids generated from patients with LBD with *SNCA* gene triplication. Single-cell RNA sequencing, combined with functional and molecular validation, identified synaptic and mitochondrial dysfunction in excitatory neurons exhibiting high expression of the *SNCA* gene, aligning with observations in the cortex of autopsy-confirmed LBD human brains. Furthermore, we screened 1280 Food and Drug Administration–approved drugs and identified four candidates (entacapone, tolcapone, phenazopyridine hydrochloride, and zalcitabine) that inhibited α-SYN seeding activity in real-time quaking-induced conversion assays with human brains, reduced α-SYN aggregation, and alleviated mitochondrial dysfunction in *SNCA* triplication organoids and excitatory neurons. Our findings establish human cortical LBD models and suggest potential therapeutic drugs targeting α-SYN aggregation for LBD.

## Teaser

*SNCA* triplication organoid model replicates Lewy body disease and advances drug discovery.

## INTRODUCTION

Aggregation of α-synuclein (α-SYN) protein is a defining characteristic of Lewy body diseases (LBDs), including Parkinson’s disease (PD), PD with dementia (PDD), and dementia with Lewy bodies (DLB) (*1*, *2*). PD is clinically characterized by bradykinesia, rigidity, postural instability, and tremor, associated with the degeneration of dopaminergic neurons in the substantia nigra with the presence of α-SYN inclusions known as Lewy bodies and Lewy neurites (*3*). PDD and DLB are characterized by progressive dementia concurrent with parkinsonian symptoms, collectively termed as Lewy body dementia, which is the second most common form of dementia after Alzheimer’s disease (AD) (*4*). Pathologically, patients with Lewy body dementia display subcortical pathological similarities to PD, but there are also diffuse Lewy bodies scattered throughout cortical brain regions, which may contribute more to the dementia symptoms (*5*). Co-occurring Lewy body pathology in the cortices has been described in more than 50% of autopsy-confirmed AD cases contributing to the severity and progression of AD symptoms (*6*, *7*). This suggests that α-SYN aggregation plays a critical role in cortical neurons associated with dementia; however, the mechanisms underlying the α-SYN pathogenesis remain unclear. Therefore, elucidating the pathophysiology of α-SYN in cortical neurons represents a crucial opportunity for understanding disease mechanisms and identifying therapeutic interventions to prevent or delay the onset and progression of dementia in both LBD and AD.

The α-SYN protein, encoded by the *SNCA* gene, is a highly abundant neuronal protein primarily found in presynaptic nerve terminals (*8*). It plays a crucial role in regulating exocytosis and the release of neurotransmitters from synaptic vesicles, thereby influencing synaptic function and neurotransmission (*8*). In pathological conditions, α-SYN undergoes a conformational change, adopting β sheet–rich structures such as oligomers and fibrils (*9*). These aggregated forms of α-SYN accumulate in Lewy bodies and Lewy neurites, leading to impaired synaptic and mitochondrial functions as well as neurotoxicity (*10*, *11*). Notably, an increased copy number of the *SNCA* gene is known to cause LBD, and the age at onset of the disease is inversely correlated with the gene dosage (*12*, *13*). These findings suggest that the concentration of α-SYN expressed within neurons plays a crucial role in the development of Lewy pathology. Further supporting this notion, human induced pluripotent stem cell (iPSC)–derived dopaminergic neurons or midbrain-like organoids generated from individuals with *SNCA* triplication have exhibited disease-related phenotypes in culture, including the accumulation of α-SYN aggregates and the loss of dopaminergic neurons (*14*, *15*). This indicates that *SNCA* triplication has an inherent ability to disrupt normal cellular function in culture, making it a potential model for studying the pathogenesis of α-SYN, including its effects on cortical neurons.

Here, we developed cortical organoid models of LBD using *SNCA* triplication iPSCs. We investigated α-SYN–related pathological changes and molecular pathways in these models, validating our findings both functionally and through comparisons with LBD human brain samples. Furthermore, we identified four Food and Drug Administration (FDA)–approved drugs that effectively inhibit α-SYN aggregation and restore mitochondrial dysfunction in iPSC-derived models with *SNCA* triplication.

## RESULTS

### *SNCA* triplication cortical organoids have increased α-SYN levels

To model cortical α-SYN pathogenesis in LBD, we generated three-dimensional (3D) cortical organoids using two control iPSC lines (Ctrl) and two *SNCA* triplication iPSC lines (SNCA Tri) derived from healthy individuals and patients with LBD, respectively (table S1). Two independent subclones from each iPSC line were used. The pluripotency and differentiation capacity of the *SNCA* triplication iPSC lines were confirmed by the expression of pluripotency markers Nanog and T-antigen-related antigen 1-60 (TRA-1-60) (fig. S1A) and their ability to differentiate into endodermal (Sox17^+^), mesodermal (Brachyury^+^), and ectodermal (Nestin^+^) cells (fig. S1B). Normal karyotypes were observed for the *SNCA* triplication iPSC lines (fig. S1C). The pluripotency and karyotype of the control iPSC lines were previously validated in published studies (*16*–*18*). Furthermore, we conducted an analysis of copy number variations (CNVs) to evaluate the *SNCA* coding region on chromosome 4, confirming the multiplication of the *SNCA* gene locus in *SNCA* triplication iPSC lines and subclones (fig. S2A). In addition, we investigated the CNV hotspot region 20q11, known for its enrichment in genes associated with pluripotency and antiapoptosis, such as DNA methyltransferase 3B (*DNMT3B*), inhibitor of DNA binding 1 (*ID1*), and BCL2-like 1 (*BCL2L1*). This region has been reported as a recurrent site of CNV during iPSC generation and differentiation, due to the genomic instability (*19*, *20*). Our analysis revealed no CNVs in this region in both control and *SNCA* triplication lines (fig. S2B), indicating that these lines do not have genomic aberrations or mutations in this critical region.

**Fig. 1.**
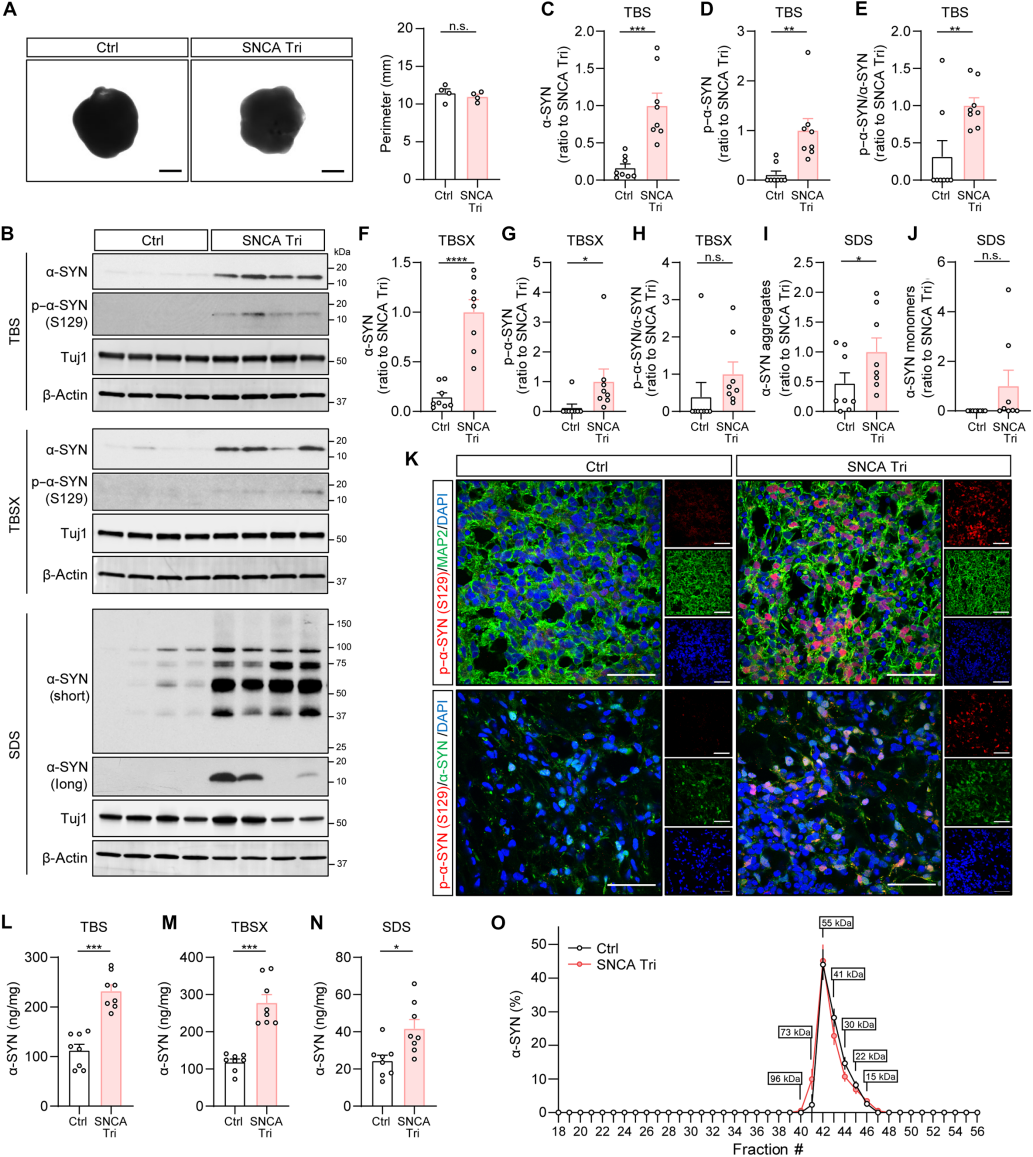
Increased levels of α-SYN in the iPSC-derived *SNCA* triplication cortical organoids. (**A**) Representative images showing the size of organoids. The perimeters of the organoids were measured and compared. Each dot on the graph represents the average perimeter of approximately 96 organoids per condition. *n* = 4 samples per group. Scale bar, 1 mm. (**B** to **J**) The levels of total α-SYN and phosphorylated α-SYN in each fraction of organoids were measured using Western blotting. Different exposure times (short and long) were applied to display and quantify the aggregated and monomeric α-SYN. Results were normalized to Tuj1 levels. *n* = 4 samples with two replicates per group. Each dot on the graph represents an individual replicate. Molecular weight markers (kilodaltons) are indicated on the right side of each blot. (**K**) The Ctrl and SNCA Tri organoids were immunostained with phosphorylated–α-SYN (S129) antibody (red) and MAP2 antibody (green, top) or total α-SYN (green, bottom). The nuclei were stained with 4′,6-diamidino-2-phenylindole (DAPI) (blue). Scale bar, 50 μm. (**L** to **N**) The levels of total α-SYN in each fraction of organoids were measured using ELISA. The experiments were conducted in duplicate. *n* = 4 samples. Each dot on the graph represents an individual replicate. (**O**) TBS-soluble organoid lysates were fractioned using SEC. The fractions ranging from #18 to #56 were collected, and the levels of α-SYN were measured using ELISA. The experiments were performed in duplicate. *n* = 4 samples per group. Data represent means ± SEM. Student’s *t* tests were used for statistical analyses. *P < 0.05; **P < 0.01; ***P < 0.001; n.s., not significant.

We then generated the cortical organoids from both control and *SNCA* triplication iPSC lines using a published protocol (*18*) and harvested the organoids after 2 months of culture. The organoids displayed expression of several neuronal cortical layer markers such as C-terminal binding protein 2 (CTIP2), Special AT-rich sequence binding protein 2 (SATB2), and T-box brain transcription factor 1 (TBR1) (fig. S3A), which are typical features of cortical organoids (*21*). We then assessed the perimeter of the organoids and found no notable difference in size between the two groups (Fig. 1A). Western blot analysis of protein extracts from buffer-soluble [tris-buffered saline (TBS)], detergent-soluble [TBS with 1% Triton X-100 (TBSX)], and insoluble fractions (SDS) showed significantly higher levels of total α-SYN in *SNCA* triplication organoids compared to control organoids (Fig. 1, B, C, F, and I). Notably, insoluble SDS fractions exhibited higher levels of high–molecular weight α-SYN (Fig. 1, B and I) in *SNCA* triplication organoids, indicating the presence of aggregated α-SYN forms. However, the levels of α-SYN monomers in the insoluble SDS fractions were similar between the two groups (Fig. 1J). Phosphorylated α-SYN levels were also elevated in *SNCA* triplication organoids in both buffer-soluble and detergent-soluble fractions (Fig. 1, B, D, E, G, and H), which was further confirmed by immunostaining (Fig. 1K). Enzyme-linked immunosorbent assay (ELISA) measurements of total α-SYN further validated the increased levels in all three fractions, with *SNCA* triplication organoids showing approximately a twofold increase compared to controls (Fig. 1, L to N).

We then measured synaptic markers [postsynaptic density (PSD95) and synaptophysin] and observed no differences between control and *SNCA* triplication organoids (fig. S3, B to D), indicating that the elevated levels of α-SYN did not lead to synaptic loss in our organoids. In addition, we investigated the levels of other proteins associated with neurodegenerative disorders, such as tau, amyloid-β (Aβ), and apolipoprotein E (APOE) (fig. S4), to determine whether *SNCA* triplication affects these proteins in addition to α-SYN. We found no differences in total tau and Aβ42 levels among the three fractions (fig. S4, A to F); however, we observed significant increases in APOE levels in the detergent-soluble fraction of *SNCA* triplication organoids compared to control organoids (fig. S4H) but no differences in the buffer-soluble and insoluble fractions (fig. S4, G and I). These findings suggest that *SNCA* triplication organoids do not alter tau or Aβ42 levels but may affect APOE metabolism.

Furthermore, in our previous study, we observed that the physiological soluble α-SYN species in human brains had a size of approximately 55 kDa (*22*). To investigate whether cortical organoids replicate this α-SYN species, we fractionated buffer-soluble organoid lysates using size exclusion chromatography (SEC) and measured α-SYN levels in each fraction using ELISA. Both control and *SNCA* triplication organoids exhibited a peak of soluble α-SYN around 55 kDa (Fig. 1O), similar to our previous observation in human brain superior temporal cortex (*22*).

Together, our results show that the iPSC-derived cortical organoids maintain the size of physiological forms of α-SYN comparable to those in human brain. *SNCA* triplication cortical organoids exhibit increased levels of α-SYN, including phosphorylated and aggregated forms.

### Single-cell RNA sequencing reveals dysregulation of crucial pathways in *SNCA* triplication organoids

**Fig. 2.**
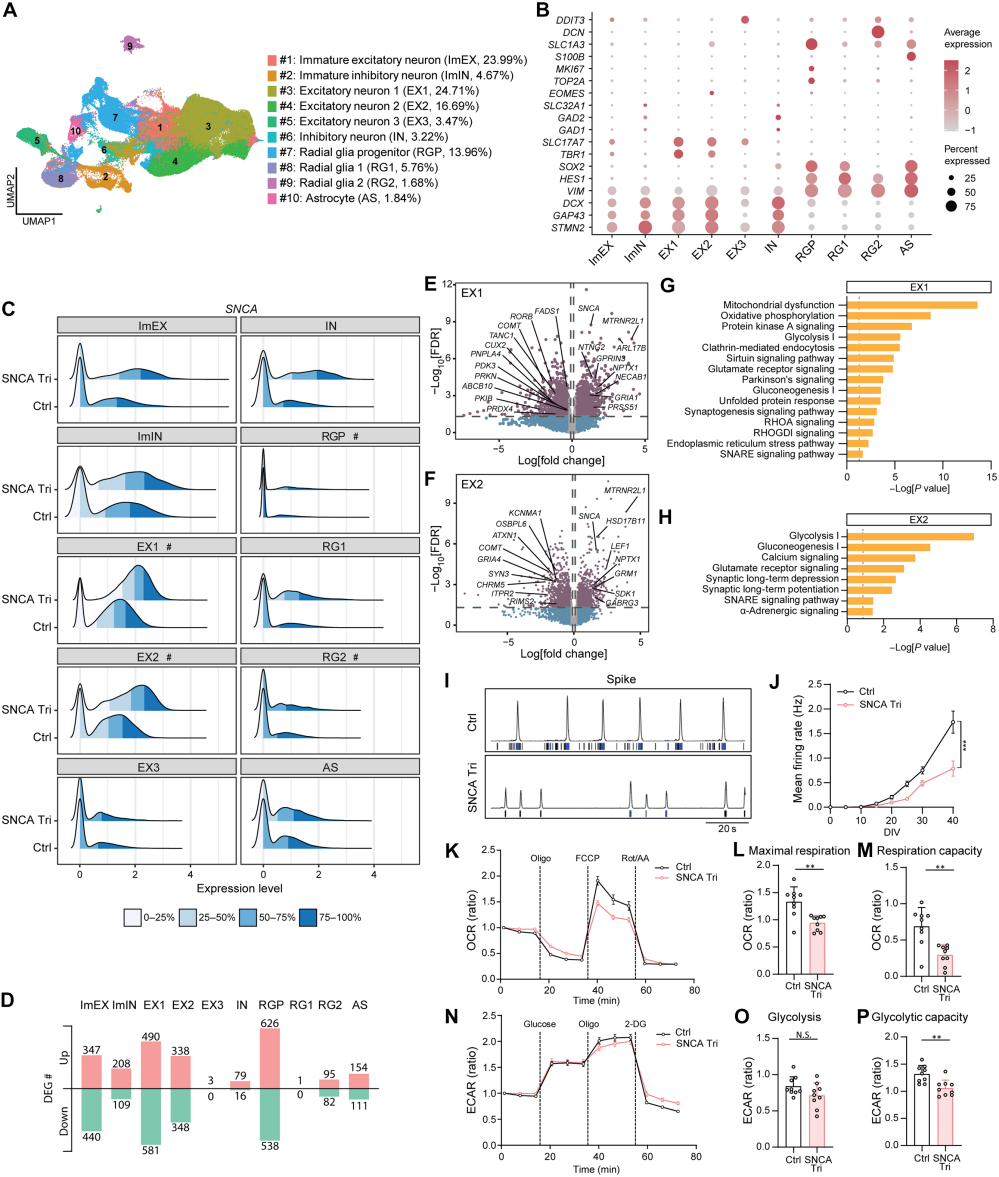
Dysregulation of crucial pathways in *SNCA* triplication organoids revealed by scRNA-seq analysis with functional validation. (**A**) UMAP plot of Ctrl and SNCA Tri organoids. The average cell population of each cluster is shown. Experiments were performed in three replicates per sample with *n* = 4 samples per group. A total of 161,920 cells were analyzed. (**B**) Dot plots of known marker genes. (**C**) Ridge plot of *SNCA* expression in each cell types. “#” indicates clusters showing significant difference. (**D**) Number of DEGs for each cell cluster. Up/Down, up-regulated/ down-regulated genes. (**E** and **F**) Volcano plots for DEGs in the EX1 (E) and EX2 (F) clusters. (**G** and **H**) Selected canonical pathways enriched by DEGs from the EX1 (G) and EX2 (H) clusters. (**I** and **J**) MEA assay. Representative spike histogram with a raster plot of electrode activity (I) and mean firing rate (J) are shown. Experiments were conducted in quadruplicate with three independent experiments. Data are presented as means ± SEM. Two-way repeated measures analysis of variance (ANOVA) was used for statistical analyses. (**K** to **P**) Seahorse XF Mito Stress test (K to M) measuring OCR (K), and maximum respiration (L) and respiratory capacity (M). Seahorse XF Glycolysis Stress test (N to P) measuring ECAR (N), glycolysis level (O), and glycolytic capacity (P). Each data point was normalized to the first data point to calculate the OCR or ECAR ratio. Experiments were conducted in triplicate with three independent experiments. Each dot represents an individual replicate, and the data are presented as means ± SEM. Student’s *t* tests were used for statistical analyses. *P < 0.05; **P < 0.01; ***P < 0.001.

To explore the molecular phenotypes of cortical organoids with *SNCA* gene triplication, we conducted single-cell RNA sequencing (scRNA-seq) analysis on dissociated organoid cells. A total of 161,920 cells were analyzed, and we identified 10 distinct cell clusters using unsupervised clustering with uniform manifold approximation and projection (UMAP) (Fig. 2A). On the basis of the expression of cell type–specific gene markers (Fig. 2B), we identified clusters representing immature excitatory neurons (ImEX; cluster #1; 23.99% of total cells) and immature inhibitory neurons (ImIN; cluster #2; 4.67% of total cells) (Fig. 2A). The majority of cells belonged to three clusters of excitatory neurons (EX1, EX2, and EX3; clusters #3, #4, and #5), accounting for 44.87% of the total cells (Fig. 2A). In addition, a small cluster of inhibitory neurons (IN; cluster #6; 3.22%) was identified (Fig. 2A). The remaining cell clusters were composed of radial glial progenitors (RGPs; cluster #7; 13.96%), radial glial cells (RG1 and RG2; clusters #8 and #9; 7.44%), and mature astrocytes (AS; cluster #10; 1.84%) (Fig. 2A). The population of the RGP cluster was significantly higher in *SNCA* triplication organoids compared to control organoids, while other cell types exhibited similar proportions (fig. S5, A and B).

To identify cell types with differential *SNCA* gene expression, we analyzed *SNCA* expression levels across each cell cluster. Our analysis revealed that the major cell clusters expressing the *SNCA* gene were EX1 and EX2, where *SNCA* level was significantly elevated in cells with *SNCA* triplication compared to control cells, supporting our expectations (Fig. 2C). It is noteworthy that although *SNCA* expression in RGP and RG2 clusters were generally low, its expression was also significantly increased in the *SNCA* triplication group compared to the control group (Fig. 2C).

We then performed analysis to identify differentially expressed genes (DEGs) between control and *SNCA* triplication organoids. We first focused on the excitatory neuron clusters (EX1, EX2, and EX3), representing the major cell type of our organoids. In EX1, we identified 490 up-regulated and 581 down-regulated genes; in EX2, there were 338 up-regulated and 348 down-regulated genes, while the EX3 cluster showed minimal changes, with only 3 up-regulated genes noted (Fig. 2D and table S2). The EX1 and EX2 clusters shared common DEGs, including up-regulated genes involved in synaptic vesicle trafficking and mitochondrial ribosome protein synthesis (*SNCA* and *MTRNR2L1*), as well as down-regulated genes associated with neurotransmitter metabolism (*COMT*) (Fig. 2, E and F). In addition, these two clusters demonstrated DEGs related to synaptic functions (up-regulated: *NECAB1*, *GRIA1*, *GRM1*, *GABRG3*, and *NPTX1*; down-regulated: *TANC1*, *CHRM5*, *ITPR2*, *GRIA4*, *SYN3*, *KCNMA1*, and *RIMS2*), mitochondrial function (down-regulated: *PRKN* and *ABCB10*), lipid metabolism (down-regulated: *FADS1*, *PNPLA4*, and *OSBPL6*), oxidative stress (down-regulated: *PRDX4*), and energy metabolism (down-regulated: *PDK3*) (Fig. 2, E and F). Furthermore, the analysis using Ingenuity Pathway Analysis (IPA) revealed that the DEGs in the clusters of excitatory neurons (EX1 and EX2) were enriched in shared pathways, including energy metabolism–related pathways such as oxidative phosphorylation, glycolysis, and gluconeogenesis, as well as synaptic pathways such as synaptogenesis signaling, soluble *N*-ethylmaleimide–sensitive factor attachment protein receptor (SNARE) signaling, and glutamate receptor signaling (Fig. 2, G and H, and table S3). Notably, the Parkinson’s signaling pathway and clathrin-mediated endocytosis signaling pathway were specifically identified in the EX1 cluster (Fig. 2G). While significant differences were observed between *SNCA* triplication and organoids in EX1 and EX2 excitatory neurons, it is important to note that high variation between iPSC lines, due to genetic and phenotypic diversity, is a well-recognized phenomenon and might limit the ability to detect small differences (*23*). As anticipated, our principal components analysis (PCA) on pseudobulk data for EX1 and EX2 clusters, revealed variability among the control organoids across different lines and subclones (fig. S5, C and D). However, the organoids with *SNCA* triplication showed similar characteristics across different lines and subclones within these subclusters (fig. S5, C and D).

In addition, within the immature excitatory neuron population (ImEX), we found 347 up-regulated genes and 440 down-regulated genes (Fig. 2D and table S2). IPA revealed that these DEGs were enriched in pathways shared with mature excitatory neurons (EX1 and EX2), including energy metabolism and synaptic functions (fig. S5E). In the smaller populations of inhibitory neurons (ImIN and IN), we observed 208 up-regulated and 109 down-regulated DEGs and 79 up-regulated and 16 down-regulated DEGs, respectively (Fig. 2D and table S2). These DEGs were notably enriched in neurotransmitter clearance and synaptic long-term depression pathways (table S3).

Furthermore, among the glial populations, the RGP subcluster exhibited the most significant differential gene expression, with 626 up-regulated genes and 538 down-regulated genes (Fig. 2D). DEGs of RGP and RG2 clusters were enriched in pathways associated with cholesterol biosynthesis and energy metabolism (fig. S5, F and G, and table S3). Last, DEGs in astrocytes (154 up-regulated genes and 111 down-regulated genes) were enriched in inflammatory pathways, including antigen presentation, interferon signaling, and phagosome maturation (fig. S5H and table S3).

Overall, these findings highlight the dysregulation of crucial pathways involved in energy metabolism and synaptic function in excitatory neurons of *SNCA* triplication organoids. In addition, the dysregulation of lipid and energy metabolism as well as inflammation-related pathways were evident in glial populations.

### *SNCA* triplication excitatory neurons display compromised neuronal activity and impaired mitochondrial function

To validate the pathway changes observed in the excitatory neuron populations identified by scRNA-seq, we differentiated control and *SNCA* triplication iPSCs into excitatory neurons [vesicular glutamate transporter 1–positive (vGLUT1^+^)] using our established protocol (*22*) (fig. S6A). Consistent with our cortical organoid model, we confirmed that at day in vitro 20 (DIV20), excitatory neurons derived from *SNCA* triplication iPSCs exhibited significantly higher levels of α-SYN in both the soluble [radioimmunoprecipitation assay (RIPA)] and insoluble (SDS) fractions (fig. S6, B to D). Moreover, the insoluble fraction of *SNCA* triplication neurons had high–molecular weight α-SYN species, indicative of protein aggregation (fig. S6B). This validation further supports our findings and highlights the relevance of *SNCA* triplication in the dysregulation of α-SYN levels and aggregation in excitatory neurons.

To validate the changes in synaptogenesis and SNARE signaling–related pathways identified from scRNA-seq, we assessed the neuronal excitability in iPSC-derived excitatory neurons. The neurons were cultured on multielectrode array (MEA) plates, and their electrical firing activity was monitored from DIV0 to DIV40 (Fig. 2, I and J). In line with the scRNA-seq data, we observed a notable reduction in individual spike activities in *SNCA* triplication iPSC-derived neurons compared to control iPSC-derived neurons (Fig. 2I). Furthermore, the mean firing rate of *SNCA* triplication excitatory neurons was significantly lower than that of control neurons, with the maximum difference observed at DIV40 (Fig. 2J). These findings provide further evidence of impaired neuronal activity in the *SNCA* triplication excitatory neurons.

In addition, we conducted experiments to assess the energy metabolism in control and *SNCA* triplication neurons at DIV20. We performed the Seahorse XF Mito Stress test to evaluate mitochondrial respiration function, which measures the oxygen consumption rate (OCR) (Fig. 2, K to M). *SNCA* triplication neurons exhibited a significant decrease in both maximal respiration (Fig. 2, K and L) and respiration capacity (Fig. 2, K and M) compared to control neurons. This indicates impaired mitochondrial function in the *SNCA* triplication neurons. Furthermore, we analyzed the glycolytic function of these neurons using the Seahorse XF Glycolysis Stress test (Fig. 2, N to P). The extracellular acidification rate (ECAR) measurements revealed a significantly lower glycolytic capacity in *SNCA* triplication neurons (Fig. 2, N and P), while there was minimal difference in glycolysis level (Fig. 2, N and O). These findings further validate our scRNA-seq results, supporting the notion of dysregulated energy metabolism in *SNCA* triplication excitatory neurons.

### *SNCA* triplication cortical organoids mimic molecular phenotypes of LBD human brains

To investigate whether *SNCA* triplication cortical organoids can replicate the molecular phenotypes associated with α-SYN pathogenesis in LBD human brains, we conducted single-nucleus RNA-seq (snRNA-seq) analysis on frozen tissue from superior temporal cortex of postmortem LBD human brains. Single nuclei were isolated from the gray matter of the superior temporal cortices, obtained from autopsy-confirmed control cases (*n* = 3) and LBD cases (*n* = 5). The LBD cases included three patients with a normal copy of the *SNCA* allele (referred to as LBD), one patient with *SNCA* duplication [referred to as LBD (SNCA Dup)], and one patient with *SNCA* triplication [referred to as LBD (SNCA Tri)] (table S4).

**Fig. 3.**
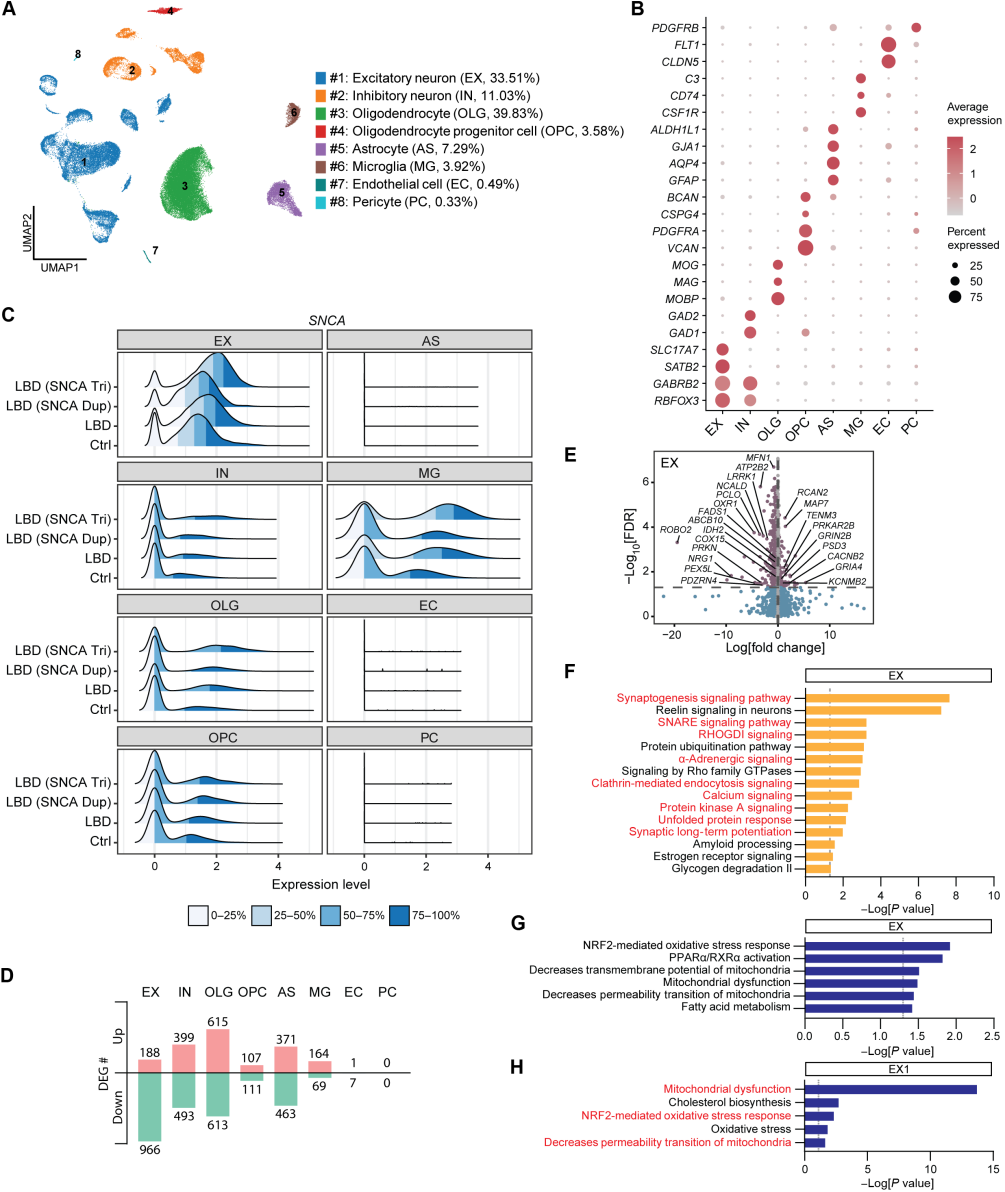
Molecular phenotypes of cortical LBD human brains overlap with *SNCA* triplication cortical organoids. (**A**) UMAP plot of Ctrl and LBD brains. Eight individual clusters were defined and annotated to distinct cell types. Average cell population of each cell cluster is indicated on the right side of the cluster name. A total of 60,644 total nuclei were analyzed. (**B**) Dot plots of known marker genes used for cell type identification. (**C**) Ridge plot of *SNCA* expression in each cell type of Ctrl, LBD, LBD (SNCA Dup), and LBD (SNCA Tri) brains. (**D**) Number of DEGs for all cell clusters in the comparison between LBD and Ctrl brains. Up-regulated genes and down-regulated genes are indicated. (**E**) Volcano plots for DEGs of LBD versus Ctrl brains in the EX cluster. (**F**) Selected canonical pathways enriched by DEGs from the EX cluster. Pathways overlapping with scRNA-seq data from *SNCA* triplication cortical organoids are indicated in red. (**G**) Top toxicity list pathways enriched by DEGs from the EX cluster of LBD human brains. (**H**) Top toxicity list pathways enriched by DEGs from the EX1 cluster of *SNCA* triplication cortical organoids. Pathways overlapping with snRNA-seq data from LBD human brains are indicated in red.

A total of 60,644 nuclei were clustered and annotated into distinct populations on the basis of the cell type–specific marker expression (Fig. 3, A and B). The major cell types identified were excitatory neurons (#1; EX; 33.51% of total cells) and oligodendrocytes (#3; OLG; 39.83% of total cells), followed by inhibitory neurons (#2; IN; 11.03% of total cells), astrocytes (#5; AS; 7.29% of total cells), microglia (#6; MG; 3.92% of total cells), oligodendrocyte progenitor cells (#4; OPC; 3.58% of total cells), endothelial cells (#7; EC; 0.49% of total cells), and pericytes (#8; PC; 0.33% of total cells), which populations did not differ between Ctrl and LBD brains (Fig. 3A and fig. S7, A and B).

Consistent with our observation from cortical organoids (Fig. 2D), *SNCA* gene expression was predominantly observed in about 90% of excitatory neurons, with the highest expression level detected in the brain of LBD (SNCA Tri) compared to LBD (SNCA Dup), LBD, and control brains (Fig. 3C; EX). A small portion of microglia also exhibited higher *SNCA* gene expression, specifically in the LBD (*SNCA* triplication) brain (Fig. 3C; MG), suggesting a potential role of *SNCA* gene in regulating microglia functions.

We then performed DEG analysis between control and LBD brains, including *SNCA* multiplication cases, to explore similarities with our cortical organoid model. The excitatory neuron cluster (EX) showed a significant number of DEGs, with 188 up-regulated genes and 966 down-regulated genes (Fig. 3D and table S5). These DEGs included genes related to synaptic function (up-regulated: *GRIA4*, *PSD3*, *CACNB2*, *KCNMB2*, and *GRIN2B*; down-regulated: *PCLO*, *NCALD*, and *ATP2B2*), mitochondrial function (down-regulated: *PRKN*, *ABCB10*, *MFN1*, and *COX15*), lipid metabolism (down-regulated: *FADS1* and *PEX5L*), oxidative stress (down-regulated: *OXR1*), and energetic metabolism (up-regulated: *PRKAR2B*; down-regulated: *IDH2*) (Fig. 3E). PCA of pseudobulk data from this excitatory neuron cluster (EX) indicated significant sample-to-sample variation but did not suggest that the two LBD cases with *SNCA* multiplication were distinct from the other three LBD cases (fig. S7C). This indicates that the observed DEGs between LBD and control samples may not be specifically driven by *SNCA* multiplication.

Through IPA canonical pathway analysis, we observed numerous overlapping pathways between the excitatory neuron clusters of organoids and human brains. These pathways were associated with synaptic function, including calcium signaling, synaptic long-term potentiation, synaptogenesis signaling pathway, SNARE signaling pathway, and clathrin-mediated endocytosis signaling. In addition, pathways such as Rho GDP-dissociation inhibitor (RHOGDI) signaling, protein kinase A signaling, α-adrenergic signaling, and unfolded protein response pathways were also shared between the excitatory neuron clusters of organoids and human brains (Figs. 2, E and F, and 3F; indicated as red text, table S6). Moreover, we identified distinct pathways enriched in human brains, including bioenergetic pathways (glycogen degradation) and protein processing pathways (e.g., protein ubiquitination pathway) (Fig. 3F). Several pathways consistently emerged across different cell clusters in human brains, such as RHOGDI signaling, protein kinase A signaling, synaptogenesis signaling pathway, signaling by Rho family guanosine triphosphatase, estrogen receptor signaling, and protein ubiquitination pathway (Fig. 3F; fig. S7, D to H; and table S6). These findings suggest the presence of common vulnerable pathways influenced by Lewy pathology across various cell types.

In addition, we conducted an IPA toxicity pathway analysis to further compare the pathways in the clusters of excitatory neuron from organoids with those in human brains. Our findings revealed that prominent pathways in both clusters of excitatory neurons were shared and associated with mitochondrial dysfunction, lipid metabolism, and oxidative stress (Fig. 3, G and H; overlapping pathways are highlighted in red).

Among the shared DEGs identified in both the excitatory neuron clusters of organoids and human brains, three genes stood out for their roles in lipid metabolism, mitochondrial function, and energy production: *FADS1* [encoding fatty acid desaturase 1 (FADS1)], *PRKN* [encoding Parkin RBR E3 ubiquitin protein ligase (Parkin)], and *ABCB10* [encoding adenosine triphosphate–binding cassette subfamily B member 10 (ABCB10)]. Notably, Parkin dysfunction, associated with an inability to regulate mitochondrial quality control, has been implicated in the pathogenesis of PD (*24*). To verify the alterations in these three genes and their corresponding proteins, we conducted quantitative polymerase chain reaction (qPCR) to assess RNA expression and Western blotting to determine protein levels in both our organoid models and autopsy-confirmed LBD postmortem human brains (tables S7 and S8). To enhance the robustness of our organoid model findings, we introduced an additional set of isogenic iPSC lines. These included a parental line with *SNCA* triplication and its isogenic control line expressing a normal copy number of the *SNCA* gene through CRISPR-Cas9 gene editing (*25*) (table S1). From these, we generated cortical organoids and cultured them for 2 months. Before validating the three shared DEGs, we initially carried out Western blotting analysis (fig. S8, A to E) and ELISA (fig. S8, F to H) to measure α-SYN levels, confirming significantly higher total α-SYN levels in the *SNCA* triplication organoids compared to the isogenic controls.

**Fig. 4.**
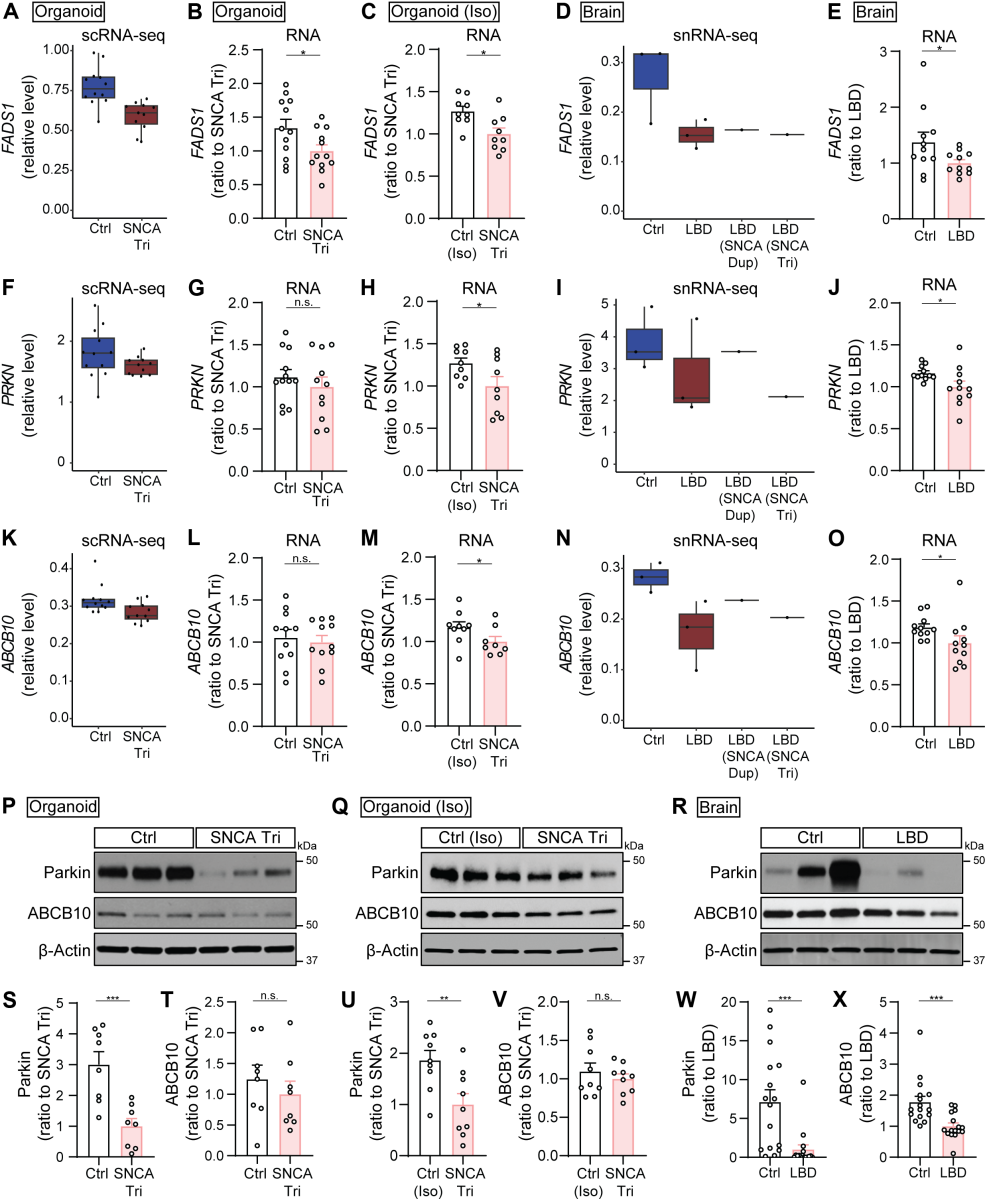
Validation of overlapping DEGs between cortical organoids and human brains. Validation of the overlapping DEGs through qPCR (A to O) and Western blotting (P to X) in control and *SNCA* triplication organoids (labeled as Organoid; B, G, L, P, S, and T), isogenic set of control (Iso) and *SNCA* triplication organoids [labeled as Organoid (Iso); C, H, M, Q, U, and V], and human brains (labeled as Brain; E, J, O, R, W, and X). (**A** to **O**) RNA levels of *FADS1* (A to E), *PRKN* (F to J), and *ABCB10* (K to O) genes. Box plots illustrate their RNA levels in the EX1 cluster of organoids (A, F, and K), and in the EX cluster of human brains (D, I, and N). Bar graphs present the RNA levels in organoids (B, G, and L), isogenic organoids (C, H, and M), and human brains (E, J, and O). (**P** to **X**) Parkin and ABCB10 levels. Representative gel images and quantification of Parkin and ABCB10 levels in TBSX fractions of organoids (P, S, and T), isogenic organoids (Q, U, and V), and human brains (R, W, and X). Results were normalized to β-actin levels. Organoids: *n* = 4 samples with three replicates (qPCR) or two replicates (Western blotting) per group; each dot on the graph represents an individual replicate. Isogenic organoids: The experiments were conducted in triplicate with three independent experiments. Each dot on the graph represents an individual replicate. Human brains: *n* = 11 samples (qPCR) or *n* = 16 samples (Western blotting) per groups; each dot on the graph represents an individual case. Data represent means ± SEM. Student’s *t* tests were used for statistical analyses. *P < 0.05; **P < 0.01; ***P < 0.001.

Aligned with the reduced *FADS1* gene expression observed in *SNCA* triplication organoids compared to controls via scRNA-seq (Fig. 4A), our qPCR analysis confirmed a decrease in *FADS1* RNA levels in *SNCA* triplication organoids from both individual iPSC lines (Fig. 4B) and isogenic lines (Fig. 4C). Similarly, the reduction of *FADS1* in human LBD brains, as indicated by snRNA-seq analysis (Fig. 4D), was validated through qPCR in a cohort of autopsy-confirmed LBD brains compared to controls (*n* = 11 cases per group; table S7 and Fig. 4E). Regarding the *PARKN* and *ABCB10* gene alterations observed in organoids via scRNA-seq (Fig. 4, F and K), these changes were validated in the isogenic set of *SNCA* triplication organoids versus controls through qPCR (Fig. 4, H and M). However, no notable differences were detected in organoids derived from individual iPSC lines (Fig. 4, G and L), likely because of greater sample-to-sample variability compared to isogenic lines. The alterations in *PARKN* and *ABCB10* genes in LBD human brains identified by snRNA-seq (Fig. 4, I and N) were also confirmed via qPCR (Fig. 4, J and O). At the protein level, we evaluated Parkin and ABCB10 in both organoids and human brains using Western blotting (Fig. 4, P to R). We observed a significant decrease in Parkin protein levels in *SNCA* triplication organoids compared to controls from individual iPSC lines (Fig. 4, P and S) and isogenic controls (Fig. 4, Q and U), as well as in a cohort of autopsy-confirmed LBD brains versus controls (*n* = 16 cases per group; table S8 and Fig. 4, R and W). However, regarding ABCB10 protein levels, notable changes were not detected in both organoid models (Fig. 4, P, Q, T, and V), despite significant alteration in brains (Fig. 4, R and X). Collectively, validating these three shared DEGs has confirmed similarities in alterations between *SNCA* triplication organoid models and LBD human brains, despite some discrepancies between RNA and protein changes in the organoid models. Overall, our data imply that the *SNCA* triplication cortical organoid model significantly recapitulates molecular endophenotype of human LBD brains at the molecular level, in particular the genes and pathways associated with synaptic functions and energy metabolisms in excitatory neurons.

### Identification of α-SYN seeding and aggregation inhibitors through screening of FDA-approved drug library

The misfolding and aggregation of α-SYN in neurodegenerative diseases involve a seeding and nucleation mechanism. Initial seeds recruit soluble α-SYN monomers, resulting in the formation of aggregates that impair mitochondrial and synaptic function, leading to neurodegeneration (*26*–*32*). Therefore, targeting the α-SYN seeding process holds promise for mitigating α-SYN pathogenesis. Using the α-SYN real-time quaking-induced conversion (RT-QuIC) assay, we had successfully replicated the seeding process and accelerated misfolding in human brain tissues with Lewy body pathology as previously reported (*22*). Building on these findings, we investigated drugs capable of inhibiting the seeding stage to intervene in α-SYN pathogenesis. We used the α-SYN RT-QuIC assay and performed high-throughput screening with an FDA-approved drug library consisting of 1280 compounds originally developed for diverse diseases. As α-SYN seeds, we used TBS brain lysates derived from superior temporal cortex of human brains with severe Lewy body pathologies, as described in our previous publication (*22*). The screening included epigallocatechin-3-gallate (EGCG) as a positive control, known for its α-SYN aggregation inhibition properties (*33*–*35*), and dimethyl sulfoxide (DMSO) as a negative control.

**Fig. 5.**
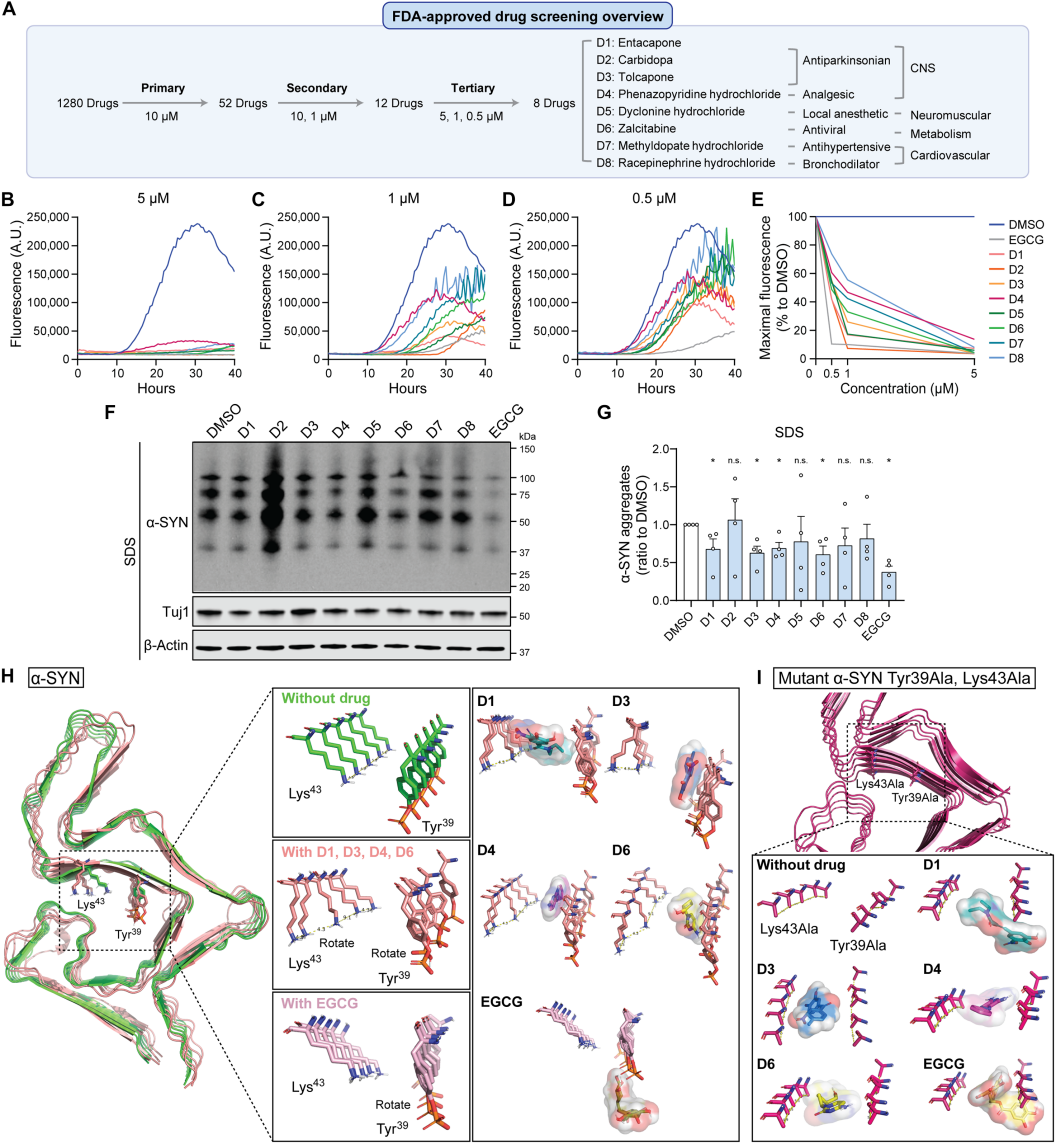
Identification of FDA-approved drug candidates inhibiting α-SYN seeding and aggregation. (**A** to **E**) Overview of FDA-approved drug screening at various concentrations to discover α-SYN aggregation inhibitors using RT-QuIC assay (A). Final eight selected drugs (D1 to D8) are listed with their original function. The aggregation curves at different concentrations (B to D) and the percentage reduction in maximal fluorescence induced by drug treatment at different concentrations (E) are shown. Negative (DMSO) and positive (EGCG) controls were included. Data represent mean values of duplicate experiments. FCCP, carbonyl cyanide *p*-trifluoromethoxyphenylhydrazone. (**F** and **G**) The drug-treated SNCA Tri organoids were lysed in RIPA and SDS buffers sequentially, and the levels of total α-SYN in the SDS fractions were measured by Western blot. DMSO and EGCG were used as controls. Four independent experiments were conducted, and data are presented as means ± SEM. Student’s *t* tests were used for statistical analyses by comparing each group with DMSO-treated group. 2-DG, 2-deoxyglucose. (**H**) The computational 3D drug-working model depicting the binding interactions between α-SYN aggregates and the drug candidates (D1, D3, D4, and D6) and EGCG at the Tyr^39^ and Lys^43^ residues. The α-SYN aggregate conformation without the drug is visualized in green, while the conformation with the drug bound is presented in pink. Representative images of α-SYN aggregate conformations, both with and without the D1 or EGCG drug, are shown to illustrate the working model at the Tyr^39^ and Lys^43^ residue sites. (**I**) The computational 3D model showing the drug’s effect was generated for mutated α-SYN, where Tyr^39^ and Lys^43^ were replaced with alanine (Tyr39Ala and Lys43Ala). The mutated α-SYN aggregate with and without drug is visualized in magenta.

Through a comprehensive screening process involving three rounds with varying drug concentrations, we identified potent inhibitors of α-SYN seeding and aggregation (Fig. 5A). In the primary screening at 10 μM, 52 drugs exhibited 100% inhibition of α-SYN seeding and aggregation, comparable to the maximal fluorescence level of positive control (EGCG). Subsequent secondary screening at 10 and 1 μM led to the selection of 12 candidate drugs that effectively inhibited α-SYN seeding at both concentrations. Tertiary screening at 5, 1, and 0.5 μM confirmed significant inhibitory effects at lower concentrations, resulting in the identification of a final set of eight drugs. These drugs demonstrated substantial inhibition, with nearly 100% efficiency at 5 μM (Fig. 5, B and E), 50% or greater at 1 μM (Fig. 5, C and E), and 30% or greater at 0.5 μM (Fig. 5, D and E) (table S9). The set includes parkinsonian drugs (D1: entacapone; D2: carbidopa; D3: tolcapone), an analgesic (D4: phenazopyridine hydrochloride), a local anesthetic for neuromuscular use (D5: pyclonine hydrochloride), an antiviral drug (D6: zalcitabine), and antihypertensive and bronchodilator drugs for cardiovascular conditions (D7: methyldopate hydrochloride; D8: racepinephrine hydrochloride).

### Selected FDA-approved drugs inhibit α-SYN aggregation and alleviate mitochondrial dysfunction in *SNCA* triplication iPSC-derived models

To assess the efficacy of the eight selected drugs in inhibiting α-SYN pathogenesis, we used our *SNCA* triplication cortical organoid model. After culturing the organoids for 1.5 months, we treated them with each drug at a concentration of 0.5 μM for 2 weeks, using DMSO as the negative control and EGCG as the positive control. After 2 months in culture, we harvested the organoids and fractionated the proteins into soluble (RIPA) and insoluble (SDS) fractions for Western blot analysis of α-SYN levels (fig. S9, A and B, and Fig. 5, F and G). The soluble fraction did not show notable differences between the DMSO-treated group and the other treatment groups (fig. S9, A and B). However, the level of aggregated α-SYN in the insoluble fraction was significantly reduced with the treatment of D1, D3, D4, D6, and EGCG compared to DMSO (Fig. 5, F and G). Electron microscopy (EM) analysis confirmed their inhibition of α-SYN fibrillization using the RT-QuIC product. The aggregates treated with DMSO exhibited long and extended α-SYN fibrils, while those treated with the four drugs and EGCG had shorter α-SYN fibrils (fig. S10A).

We next conducted computational modeling studies to elucidate the binding mechanism of these four potential drug candidates that inhibit α-SYN aggregation. Using Xtra Precision Docking Mode (Glide), we performed docking simulations of the drug candidates and EGCG with α-SYN aggregates to assess the strength of their interactions (fig. S11). Our findings revealed that these four drugs effectively inhibit α-SYN assembly by inducing conformational changes in two key amino acids, Lys^43^ and Tyr^39^, within the binding site (Fig. 5H and fig. S11, A to D). Specifically, the binding of drugs D1, D3, D4, and D6 altered the stacking arrangement of Lys^43^ side chains, shifting them by a 45° angle from their normal position in the unliganded state of α-SYN aggregates. This disruption in the network of interactions and the regular pattern within the α-SYN aggregates potentially hinders further α-SYN seeding. In addition, the drug interactions caused a shift in the phosphorylated Tyr^39^ ring of α-SYN aggregates. However, EGCG did not fit into the same binding site; instead, it attached to Tyr^39^ and other residues within the α-SYN aggregates and altered the angle of Tyr^39^ but not affecting Lys^43^, likely because of EGCG’s larger size compared to other drug candidates (Fig. 5H and fig. S11E). To validate the specificity of our findings, we tested two negative drug candidates, mepenzolate and metixene, which had shown no inhibitory effects on α-SYN aggregation during the primary drug screening using RT-QuIC. We confirmed that the negative control drugs did not exhibit any binding affinity or induce alterations in the side chains (fig. S11, F and G). To further test the roles of two key residues, Lys^43^ and Tyr^39^, in drug interactions that inhibit α-SYN aggregation, we carried out docking simulations with drug candidates and EGCG on α-SYN mutants where Lys^43^ and Tyr^39^ were substituted with alanine (Fig. 5I). With these mutations, D1, D3, D4, D6, and EGCG could not induce rotation in the Lys43Ala or Tyr39Ala residues, leading to no change in the conformation of α-SYN aggregates necessary to prevent further accumulation. Collectively, these results provide insights into an intriguing mechanism whereby drug binding in a specific region of the α-SYN aggregate, involving Lys^43^ and Tyr^39^ residues, induces rotamer shifts in specific side chain amino acids. This disruption affects both inter- and intraprotein interactions, as well as the propensity of α-SYN to form aggregate stacks.

**Fig. 6.**
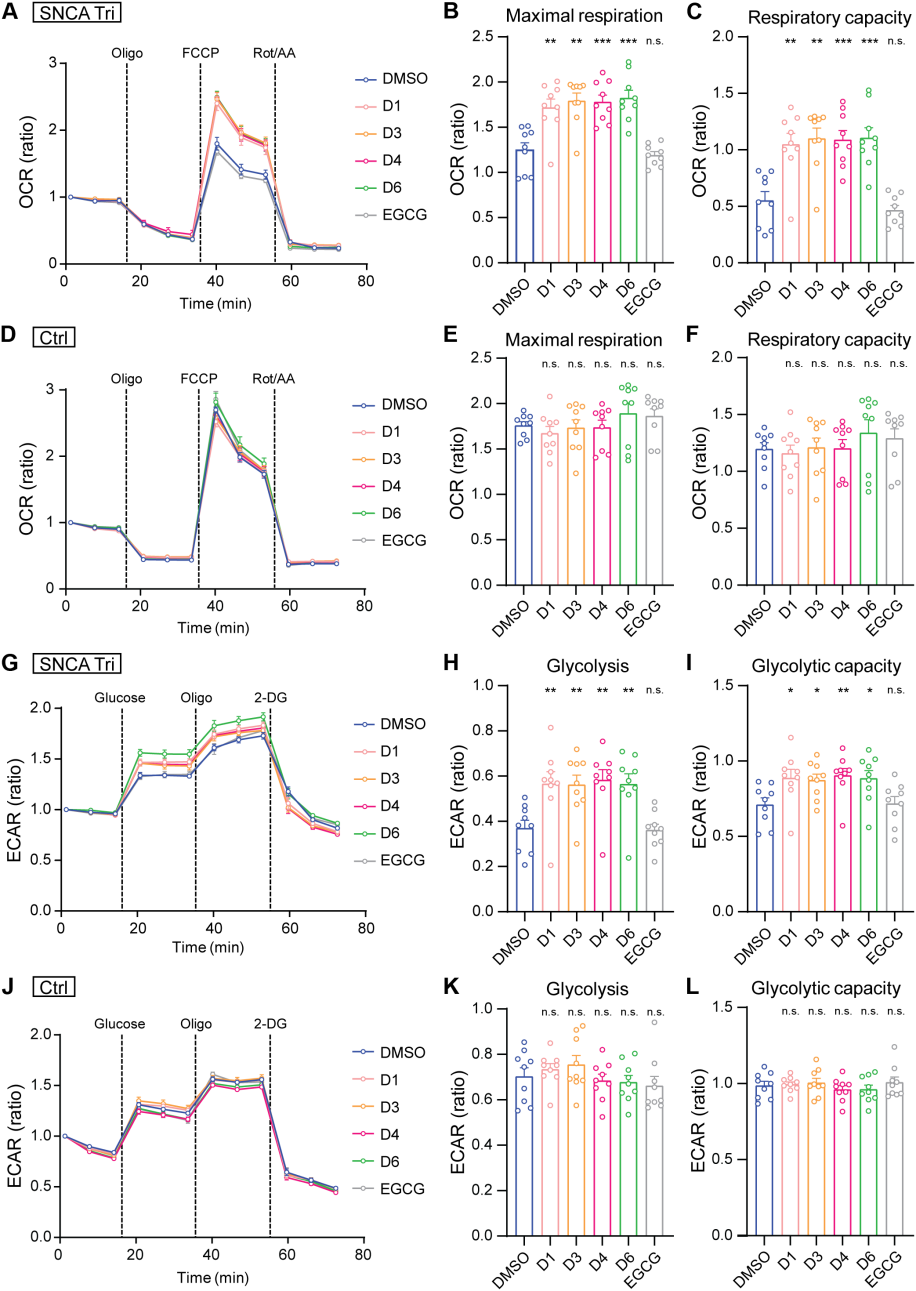
Effects of drug treatment on alleviating mitochondrial dysfunction in *SNCA* triplication iPSC-derived models. (**A** to **L**) SNCA Tri and Ctrl neurons were treated with the final four drug candidates (D1, D3, D4, and D6) at 0.5 μM from DIV7. Seahorse assay was performed at DIV20. Seahorse XF Mito Stress test (A to F) measured OCR, comparing DMSO- to drug-treated SNCA Tri and Ctrl neurons. Maximum respiration (B and E) and respiratory capacity (C and F) were analyzed. Seahorse XF Glycolysis Stress test (G to L) measured ECAR, comparing DMSO- and drug-treated neurons. Glycolysis level (H and K) and glycolytic capacity (I and L) were determined. EGCG was used as a reference. Each data point was normalized to the first data point to calculate the OCR or ECAR ratio. Three independent experiments were conducted in triplicate, and each dot represents an individual replicate. Data are shown as means ± SEM. Student’s *t* tests were used for statistical analyses by comparing each group with DMSO-treated group. *P < 0.05; **P < 0.01; ***P < 0.001.

Furthermore, we conducted experiments to evaluate the ability of the four drug candidates (D1, D3, D4, and D6) to mitigate α-SYN–related toxicity. Considering the identified bioenergetic dysfunction in excitatory neurons with *SNCA* triplication (Fig. 2, K to P), we investigated the potential of these four drugs candidates to enhance energy metabolism in *SNCA* triplication neurons (Fig. 6, A to L). Starting from DIV7, *SNCA* triplication and control excitatory neurons were treated with each drug at a concentration of 0.5 μM. At DIV20, Seahorse XF Mito Stress and Seahorse XF Glycolysis Stress tests were conducted to assess mitochondrial respiration function and glycolytic function, respectively (Fig. 6, A to L). Notably, *SNCA* triplication neurons treated with the four drugs showed a significant increase in maximal respiration level (Fig. 6, A and B) and respiration capacity (Fig. 6, A and C) compared to DMSO-treated neurons. In addition, the four drugs enhanced glycolysis levels (Fig. 6, G and H) and glycolytic capacity (Fig. 6, G and I) in *SNCA* triplication neurons compared to the DMSO control, whereas EGCG had no effect on any metabolic recovery. In contrast, control neurons showed no changes in mitochondrial or glycolytic functions following drug treatment (Fig. 6, D to F and J to L). This suggest that the observed impacts of the drugs on mitochondrial functions in *SNCA* triplication neurons are probably associated with their ability to inhibit α-SYN aggregation.

We then investigated whether treatment with these drugs in organoids could improve the expression of genes associated with lipid metabolism, mitochondrial function, and bioenergetic pathways, which were down-regulated in *SNCA* triplication organoids and LBD human brains (Fig. 4). Using qPCR, we assessed the RNA levels of *FADS1*, *PRKN*, and *ABCB10* in *SNCA* triplication organoids after 2 weeks of drug treatment (fig. S12, A to C). Notably, D6 induced an increase in the levels of *FADS1*, *PRKN*, and *ABCB10*, D1 specifically enhanced PRKN levels, whereas D3 and D4 showed no impact on the expression of these genes (fig. S12, A to C). Together, these findings indicate that the FDA-approved drugs—entacapone, tolcapone, phenazopyridine hydrochloride, and zalcitabine—not only inhibit α-SYN aggregation but also alleviate mitochondrial dysfunctions in the *SNCA* triplication organoids and excitatory neurons.

## DISCUSSION

In this study, we developed a cortical LBD model using cerebral organoids derived from *SNCA* triplication iPSCs and explored its therapeutic applications. The *SNCA* triplication organoid model had increased levels of phosphorylated and aggregated α-SYN compared to control organoids. The *SNCA* triplication cortical organoids closely resembled the cortex of human LBD brains in terms of α-SYN size distribution and predominant *SNCA* gene expression in excitatory neurons and shared molecular pathway alterations involving impaired synaptic and mitochondrial functions, as revealed by biochemical assays, functional tests, and scRNA-seq and snRNA-seq analyses. Moreover, we identified four FDA-approved drugs—entacapone, tolcapone, phenazopyridine hydrochloride, and zalcitabine—that effectively inhibited α-SYN seeding in human brain tissues and demonstrated the ability to inhibit α-SYN aggregation and restore mitochondrial function in the cortical LBD iPSC model. These findings highlight the translational potential of this model system for studying pathobiology of LBD and discovering novel therapies.

The pathogenesis of α-SYN in dopaminergic neurons in LBD has been extensively studied (*36*, *37*). On the other hand, the specific vulnerabilities and disease processes in cortical neurons, which are crucial for the dementia in LBD, have not been thoroughly explored. iPSC models offer the advantage of generating human cellular models from patients with susceptible genetic backgrounds. The iPSC-derived 3D organoids provide a unique opportunity to investigate complex human diseases with architectural and physiological complexity (*38*). Triplications of the *SNCA* gene are associated with early-onset LBD, characterized by widespread α-SYN aggregates in cortical and subcortical brain regions (*39*–*41*). Although *SNCA* triplications are rare (*42*), their robust phenotype and rapid disease progression make them a valuable resource for advancing our understanding of LBD and developing targeted interventions. In our study, iPSC-derived cortical organoids with *SNCA* triplications successfully recapitulated key features observed in the cortex of LBD, including increased levels of phosphorylated and aggregated α-SYN, impaired synaptic functions, and disrupted energy metabolism in excitatory neurons. These findings are consistent with alterations observed in excitatory neurons in the anterior cingulate cortex of LBD brains by snRNA-seq (*43*). Increased α-SYN accumulation and bioenergetic deficits have also been observed in *SNCA* triplication iPSC-derived dopaminergic neurons and midbrain-like organoids (*14*, *15*, *44*, *45*), as well as in *SNCA* triplication iPSC-derived cortical spheroids (*46*), suggesting a potential common pathogenesis in both neuronal types (*11*). Cortical organoids may also be susceptible to mitochondrial damage that mimics α-SYN pathogenesis in the brain, making them a valuable model for research on LBD.

While the *SNCA* triplication cerebral organoid model offers valuable insights into LBD pathology, it has limitations. One is the relative immaturity and lack of cellular diversity in the organoids, which do not include key cell types such as oligodendrocytes and microglia. Moreover, we had limited numbers of *SNCA* triplication iPSC lines because of its rarity. Furthermore, differences among iPSC lines due to their distinct genetic backgrounds may influence downstream molecular and functional studies. To overcome this challenge, it is essential to conduct experiments with *SNCA* triplication organoids and their isogenic control counterparts to ensure the reliability of these findings. Furthermore, while the organoids showed increased levels of phosphorylated α-SYN and higher–molecular weight aggregates of α-SYN, they did not develop the Lewy bodies typically seen in human LBD brains. In addition, in our study, we were unable to amplify aggregated α-SYN by the RT-QuIC assay in organoids cultured for 2 months, likely because of their immaturity, which lacks the robust seeding capability often seen in more developed stages. Future studies could address these limitations by extending the culture period and introducing other cell types into the model system. Another limitation is that the *SNCA* triplication iPSCs and LBD human brains were from male patients only, reflecting the higher prevalence of LBD cases in men (*44*). Nevertheless, it would be valuable for future investigations to explore the mechanisms underlying sex differences in LBD and to include female samples to better understand disease in both sexes.

LBD and related disorders have a significant impact on the quality of life for affected individuals (*47*, *48*), yet there is a lack of effective therapies targeting α-SYN aggregation and toxicity. In our study, we used the RT-QuIC assay using α-SYN seeds derived from LBD human brains (*22*) and *SNCA* triplication organoid models to screen 1280 FDA-approved drugs. The four promising drug candidates include entacapone and tolcapone, both of which are selective inhibitors of catechol-*O*-methyltransferase (COMT) and are commonly used in the treatment of PD, typically in combination with levodopa and carbidopa (*49*). These two drugs have also shown antiamyloidogenic properties against α-SYN and Aβ42 (*50*). We consistently observed their strong inhibitory effect on α-SYN aggregation, supporting a potential therapeutic benefit. The other two drug candidates that inhibit α-SYN aggregation are phenazopyridine hydrochloride, used to alleviate urinary urgency and known to affect autophagy and neuronal differentiation at the cellular level (*51*, *52*), and zalcitabine, an antiviral medication.

Although the exact roles of these two drug candidates in LBD are yet to be established, their ability to inhibit α-SYN seeding in RT-QuIC assays with human brains, as well as endogenous α-SYN fibrillization and aggregation in organoids, represents a significant step forward in targeting α-SYN aggregation and mitigating mitochondrial dysfunction. In addition, these drug candidates can be further tested using other cell-based α-SYN seeding assays, such as the fluorescence resonance energy transfer assay. Nonetheless, our findings provide a promising framework for the development of therapeutic strategies for LBD and related disorders. Our simulation model demonstrated the potential binding of four drug candidates to the α-SYN aggregate residues Lys^43^ and Tyr^39^ in preventing the accumulation of α-SYN aggregates; conversely, EGCG attached to Tyr^39^ and additional residues, playing a distinct role in preventing α-SYN aggregation. This suggests that the drug candidates and EGCG may use different mechanisms to alleviate mitochondrial dysfunction, warranting further investigation. Moreover, before advancing these substances to clinical trials, essential aspects need to be addressed, including their capacity to cross the blood-brain barrier, biodistribution across various cell types, safety evaluations, dosage refinement, and detailed understanding of their mechanisms of action.

In summary, we developed a cortical LBD model using *SNCA* triplication iPSC-derived organoids. These organoids recapitulated key LBD features in excitatory neurons. By combining this model with the RT-QuIC assay, we identified FDA-approved drugs that target α-SYN aggregation. Our findings provide insights into LBD pathology and offer a potential therapeutic approach for preventing α-SYN aggregation in excitatory neurons.

## MATERIALS AND METHODS

### Generation of iPSCs from human skin fibroblasts

Human skin biopsies from two normal individual and two *SNCA* triplication patients with LBD were obtained with patient consent from Mayo Clinic under Institutional Review Board protocols approved by the Mayo Clinic Institutional Review Board (no. 09-003803). Two independent subclones from each of the four individual iPSC lines were used to generate organoids for analysis. The *SNCA* genotype were confirmed by TaqMan Copy Number Assays. iPSCs were generated as previously described (*16*, *18*). Cells were cultured in fibroblast medium containing 10% fetal bovine serum (Gemini Bio-Products). Three episomal vectors were electroporated into the fibroblasts using the Human Dermal Fibroblast Nucleofector Kit (Lonza) (*53*, *54*). Fibroblasts were transfected with 3 μg of expression plasmid mixtures with 100 μl of transfection reagents and then plated onto a 100-mm Matrigel (Corning)–coated dish. Fibroblast medium was replaced with TeSR E7 complete medium (STEMCELL Technologies) after 5 days. Daily medium change was performed for 3 to 4 weeks. iPSC colonies were isolated and expanded in complete mTeSR1 medium for further characterization. The iPSC colonies were passaged using dispase (STEMCELL Technologies) and subjected to rock inhibitor Y27632 (STEMCELL Technologies) treatment for the first 24 hours. Karyotyping of the iPSC clones was performed by Mayo Clinic Cytogenetics Core. To validate the key findings derived from these organoids from four individual iPSC lines, a set of isogenic iPSC lines was purchased from American Type Culture Collection, including one parental *SNCA* triplication iPSC line and an isogenic control iPSC line with a normal copy of the *SNCA* genes achieved through CRISPR-Cas9 gene editing (table S1).

### Trilineage differentiation of human iPSCs

Three–germ layer differentiation was used to confirm the pluripotency of iPSCs using the STEMdiff Trilineage Differentiation kit (STEMCELL Technologies) according to the manufacturer’s instructions with some modification (*16*, *55*). When iPSCs were confluent, they were passaged using Accutase (STEMCELL Technologies) and plated onto a Matrigel-coated 12-well plate. The dissociated iPSCs were subjected to differentiation with specific differentiation media into mesoderm and endoderm lineages for 5 days or an ectoderm lineage for 7 days. Differentiation was assessed by immunostaining for germ layer–specific markers (endoderm: SOX17; mesoderm: Brachyury; ectoderm: Nestin/Sox2).

### Immunofluorescence staining

The cells were fixed in 4% paraformaldehyde, permeabilized in 0.2% Triton X-100, then blocked in 2% bovine serum albumin for 1 hour, and incubated overnight with the following primary antibodies: Nanog (Cell Signaling Technology, 1: 300), TRA-1-60 (Abcam, 1:300), Sox17 (Abcam, 1: 300), Brachyury (R&D Systems, 1: 300), Nestin (Abcam, 1:500), CTIP2 (Abcam, 1:200), SATB2 (Abcam, 1:200), TBR1 (Abcam, 1:200), MAP2 (microtubule-associated protein 2; Abcam, 1:1000), α-synuclein (BioLegend, 1:300), phospho–α-synuclein (Cell Signaling Technology, 1:200), TUJ1 (β-III tubulin, Sigma-Aldrich, 1:500), and vGLUT1 (Synaptic Systems, 1:100). Cells were then incubated with Alexa Fluor–conjugated secondary antibodies for 2 hours at room temperature (Invitrogen, 1:400). After 4′,6-diamidino-2-phenylindole staining, images were scanned using a confocal microscope.

### CNV analysis

#### DNA extraction

Genomic DNA was extracted from iPSC lines using the Promega Wizard Genomic DNA Purification Kit (Promega Corporation) following the manufacturer’s instructions. Briefly, each sample was lysed using Nuclei Lysis Solution followed by the addition of RNase Solution. The samples were then incubated for 15 min at 37°C. After allowing samples to cool to room temperature, Protein Precipitation Solution was added, vortexed, and chilled on ice for 5 min. Samples were centrifuged at 16,000*g* for 4 min. Supernatant was transferred to a fresh tube containing isopropanol and mixed gently by inversion followed by centrifugation at 16,000*g* for 1 min. The pellet was washed using 70% ethanol followed by another centrifugation at 16,000*g* for 1 min. The ethanol was aspirated allowing the pellet to air dry for 15 min. The DNA was rehydrated using DNA Rehydration Solution.

#### Infinium Global Screening Array

All DNA samples were sent to the Mayo Clinic Genomics Core to be genotyped using the Infinium Global Screening Array and iScan System (Illumina). The CNV calling was performed using Genome Studio v2.0 and cnvPartition v3.2.0 (Illumina).

### Cerebral organoid culture

STEMdiff Cerebral Organoid Kit (STEMCELL Technologies) was used to generate cerebral organoids following the manufacturer’s instructions as we previously reported (*18*). Briefly, on day 0, iPSC colonies were dissociated into single-cell suspension with Accutase, where 15,000 cells were seeded into a U-bottom ultralow-attachment 96-well plate in embryoid body (EB) formation media (medium A) supplemented with 10 μM Y-27632. On days 2 and 4, additional 100 μl of medium A was added per well. On day 5, EBs were moved to 48-well low-attachment plates in neural induction medium (medium B) and left for an additional 3 to 5 days. EBs were further embedded into 20 μl of Matrigel and cultured in neural expansion medium (medium C + D) for 3 days in six-well low-attachment plates for organoid formation. Last, organoids were transferred to 10-cm dishes and moved to an orbital shaker for further culture in neural culture medium (medium E). After 4 weeks, medium E was replaced with neuronal maturation medium consisting of the following: Dulbecco’s modified Eagle’s medium (DMEM)/F12 + Neurobasal Medium (1:1) supplemented with N2, B27, brain-derived neurotrophic factor (BDNF) (20 ng/ml), glial cell line–derived neurotrophic factor (GDNF) (20 ng/ml), ascorbic acid (200 μM), and dibutyryl cyclic adenosine monophosphate (dbcAMP) (100 nM) (Sigma-Aldrich). Cerebral organoids were harvested after 2 months of differentiation, and three to four organoids pooled for immunostaining and biochemical analysis. For drug treatment, three organoids were pooled and treated with each drug at 0.5 μM concentration every 3 to 4 days for 2 weeks. The drug-treated organoids were harvested at 2 months for biochemical analysis.

### Protein extraction from organoid

Organoids were subjected to three-step sequential protein extractions using TBS buffer, detergent-containing buffer (TBSX), and 2% SDS buffer (SDS) to obtain the buffer-soluble (TBS), detergent-soluble (TBSX), and insoluble (SDS) proteins, respectively. Briefly, organoids were homogenized in ice-cold TBS buffer containing a protease inhibitor cocktail (Roche) and a phosphatase inhibitor (Roche), sonicated, and incubated at 4°C for 30 min with end-over-end agitation. The supernatant was collected after centrifugation at 100,000*g* for 30 min at 4°C as TBS fraction. The residual pellet was rehomogenized in TBSX buffer with protease and phosphatase inhibitors, sonicated, incubated at 4°C for 30 min with end-over-end agitation, and centrifuged as above to obtain supernatant as the TBSX fraction. The pellet was resuspended in 2% SDS buffer, sonicated, and incubated for 30 min at room temperature. With the same centrifugation as above, the insoluble supernatant (SDS fraction) was collected for further assays. Drug-treated organoids were subjected to two-step sequential protein extractions instead using RIPA buffer (Thermo Fisher Scientific) and 2% SDS buffer (SDS) to obtain the soluble (RIPA) and insoluble (SDS) proteins using the same centrifugation method, respectively.

### Western blotting

The proteins were resolved by SDS–polyacrylamide gel electrophoresis, and transferred to polyvinylidene difluoride membranes, which were subsequently blocked using 5% milk in phosphate-buffered saline (PBS). After blocking, proteins were detected with a primary antibody overnight at 4°C. The next day, membranes were washed and probed with horseradish peroxide (HRP)–conjugated secondary antibody and developed with enhanced chemiluminescence imaging. The primary antibodies were as follows: anti–α-synuclein (BioLegend, 1:1000), phospho–α-synuclein (Cell Signaling Technology, 1:200), PSD95 (Abcam, 1:1000), synaptophysin (Abcam, 1:1000), Parkin (Abcam, 1:1000), ABCB10 (Abcam, 1:1000), TUJ1 (β-III tubulin, Sigma-Aldrich, 1:500), and anti–β-actin (Sigma-Aldrich, 1:2000).

### Size exclusion separation of soluble proteins in TBS fraction of the organoids

TBS-soluble organoid lysates were fractionated by SEC with AKTA fast protein liquid chromatography (GE Healthcare) and tandem Superose 6, 10/300 GL columns (GE Healthcare) in phosphate buffer containing 50 mM sodium phosphate (pH 7.4), 150 mM NaCl, and 1 mM EDTA at a flow rate of 0.3 ml/min and 800 μl per fraction. TBS fractions (500 μl per sample) from three to four pooled organoids were subjected to SEC.

### Quantification of α-SYN, tau, Aβ42, and APOE by ELISA

The organoid lysates from TBS, TBSX, and SDS fractions, as well as the SEC-separated TBS fractions, were used to assess the amounts of α-SYN, APOE, tau, and Aβ42 by ELISA. The amount of α-SYN was measured using a commercially available sandwich ELISA using a mouse monoclonal capture antibody (Anaspec) based on the manufacturer’s protocol as we previously reported (*22*). All samples and standards were assayed in duplicates and then averaged. The measurements of APOE, tau, and Aβ42 in each fraction were performed using the ELISA protocols described previously (*22*). Briefly, for APOE ELISA, WUE-4 capture antibody (Novus) and biotin-conjugated detection antibody (K74180B, Meridian Life Science) were used. Aβ42 levels were measured using monoclonal antibody (2.1.3) and an HRP-conjugated detection antibody (Ab5). Aβ antibodies were produced in-house (*56*). The levels of tau were determined by ELISA using a monoclonal tau antibody (HT7; Thermo Fisher Scientific) as a capture antibody and a biotin-conjugated tau antibody (BT2; Thermo Fisher Scientific) as a detection antibody.

### Single-cell isolation of cerebral organoids for RNA library preparation

Two independent subclones from two control lines and two *SNCA* triplication lines were used to generate organoids. Each subclone per line had three replicates, with three to four organoids pooled together to reduce variability between samples (table S10). Single cells from these organoids were isolated using Neural Tissue Dissociation Kits (Miltenyi Biotec) according to the manufacturer’s instructions following the study previously reported (*57*). RNA library preparation was performed on isolated organoid samples using the chromium platform (10x Genomics) with the Chromium Next GEM Single Cell 3′ v3.1 kit, using a targeted input of 6000 cells per sample. In brief, gel beads in emulsions (GEMs) were generated on the sample chip in the Chromium controller. Barcoded cDNA was extracted from the GEMs using Post GEM-RT Cleanup and amplified for 12 cycles. Amplified cDNA was fragmented and subjected to end-repair, poly-A tailing, adaptor ligation, and 10×-specific sample indexing following the manufacturer’s protocol. Libraries were quantified using the TapeStation (Agilent) analysis and then sequenced on a HiSeq 4000 instrument (Illumina) targeting a depth of 25,000 to 50,000 reads per cell.

### scRNA-seq data analysis

Single-cell sequence preprocessing was performed using the standard 10x Genomics Cell Ranger Single Cell Software Suite v.6.1.1. Briefly, raw sequencing data were demultiplexed, aligned to the human genome GRCh38-2020-A, and the reads aligned to each gene were counted. Cells were removed from each organoid sample if the number of features and the number of unique molecular identifier (UMI) counts both exceeded the 99th quantile calculated per sample. In addition, cells were removed if the percentage of mitochondrial gene counts exceeded 25% of all counts per cell or if the number of features per cell was less than 250. Initial dimensionality reduction, clustering, and visualization showed that sample variation was largely due to variation between cell line subclones. Thus, the counts were normalized with Seurat’s SCTransform (SCT) function, separately by cell line subclone, and the SCT Pearson residuals were further adjusted by the cellular mitochondrial and ribosomal gene percentage (by setting the vars.to.regress parameter of SCT).

#### Cell clustering and annotation

The samples were then integrated by subclone using the Seurat version 3 pipeline, choosing one of the subclones (MC0192 #4) as reference batch for computational efficiency. Samples were visualized with Louvain clustering and UMAP visualization. Following integration, one of the samples (#13) was removed (table S10) because of a large abundance of low-quality cells containing a mixture of cluster markers. Samples were again integrated following the same procedure as before. Cluster markers were computed using Seurat’s FindAllMarkers function on the Pearson residuals of SCT-normalized data (scale.data slot of SCT assay). Cell clusters were manually annotated as in the study by Tanaka *et al*. [methods and supplementary figure S1B in (*58*)]. Each cluster was subsetted and individually integrated as previously described to identify and remove clusters of multiplets and other contaminating cell clusters. Following this step, cells were once again integrated together using the above pipeline. Again, following Louvain clustering, marker genes were computed as above, and cell clusters were manually annotated on the basis of marker gene expression.

#### Differential gene expression analysis

DEGs were computed with a combination of both pseudobulk and cell-wise differential expression methods. For pseudobulk differential expression, cells were subset by cell type and their raw RNA counts summed per replicated sample. These were analyzed following the edgeR quasi-likelihood pipeline: Genes were filtered with edgeR’s filterByExpr function; normalization factors were computed with calcNormFactors; and sample gene expression was modeled with Genotype and flow cell. For cell-wise differential expression, the MAST two-part hurdle model was applied onto cells of each cell type, modeling cells with genotype and the additional covariates of cellular detection rate, mitochondria percentage, and subclone. A gene was considered differentially expressed if the false discovery rate (FDR) was less than 0.05 for both tests, if the estimated fold changes were the same direction in both tests, and if absolute log_2_ fold change (log_2_FC) was greater than 0.2 in pseudobulk method and coefficient was greater than 0.04 in cell-wise method. The DEGs were used as inputs for IPA.

### Culture and differentiation of iPSC-derived excitatory neurons

Human iPSCs were differentiated into excitatory neurons as previously described (*22*). Briefly, iPSCs were cultured in commercial neural induction medium (STEMCELL Technologies) according to the manufacturer’s instructions with some modifications. To initiate neurosphere formation, iPSC clumps were cultured in neural induction medium for 5 to 7 days in AggreWell (STEMCELL Technologies). To induce neural rosette formation, the neurospheres were seeded onto Matrigel-coated dishes and cultured in neural induction medium for another 5 to 7 days. Neural rosettes were isolated as a single-cell suspension and replated onto Matrigel-coated dishes in neural induction medium. To differentiate into neural progenitor cells (NPCs), the medium was replaced to NPC medium (STEMCELL Technologies) and cultured for additional 10 to 14 days. NPCs were amplified, and frozen stocks were made for further experiments. For neuronal differentiation, NPCs were seeded on poly-l-ornithine (Sigma-Aldrich)– and laminin (Sigma-Aldrich)–coated plates in NPC medium. The following day, the media was replaced to neuronal differentiation medium [DMEM/F12 and Neurobasal Medium (1:1) supplemented with N2, B27, BDNF (20 ng/ml), GDNF (20 ng/ml), NT3 (10 ng/ml), insulin-like growth factor (IGF) (10 ng/ml), ascorbic acid (200 μM) (all from STEMCELL Technologies), and dbcAMP (100 nM) (Sigma-Aldrich)] to differentiate NPCs into neurons for indicated days. For drug treatment, neurons at DIV7 were treated with each drug at 0.5 μM concentration every 2 to 3 days. The drug-treated neurons were harvested at DIV20 for biochemical analysis.

### Protein extraction from iPSC-derived neurons

iPSC-derived neurons were subjected to two-step sequential protein extractions using RIPA buffer and 2% SDS buffer (SDS) to obtain the soluble (RIPA) and insoluble (SDS) proteins, respectively. Briefly, the cells were homogenized in ice-cold RIPA buffer containing a protease inhibitor cocktail (Roche) and a phosphatase inhibitor (Roche), vortexed, and incubated at 4°C for 30 min. The supernatant was collected after centrifugation at 13,000 rpm for 30 min at 4°C as RIPA fraction. The residual pellet was rehomogenized in SDS buffer with protease and phosphatase inhibitors, sonicated, incubated at room temperature for 30 min with agitation, and centrifuged at 100,000*g* for 30 min at room temperature to collect supernatant (SDS fraction).

### Multielectrode array

iPSC-derived excitatory neurons were plated as droplets in poly-l-ornithine (Sigma-Aldrich)– and laminin (Sigma-Aldrich)–coated wells of a CytoView MEA 24-well plate (Axion BioSystems) according to the manufacturer’s instructions. After 4 hours of incubation in 37°C, droplets were flooded with neuron media composed of DMEM/F12 and Neurobasal Medium (1:1) supplemented with N2, B27, BDNF (20 ng/ml), GDNF (20 ng/ml), NT3 (10 ng/ml), IGF (10 ng/ml), ascorbic acid (200 μM) (all from STEMCELL Technologies), and dbcAMP (100 nM) (Sigma-Aldrich). The neurons were maintained in the media up to DIV40 with recording sessions every 5 to 10 days. All extracellular recordings were performed using the Axion Maestro Pro MEA system (Axion Biosystems). Spontaneous neural activity was recorded for 30 min, and bursts were detected at each electrode using an interspike interval threshold. Electrodes were defined as active if neuronal firing occurred at a minimal rate of 5 spikes/min. For MEA data analysis, only wells containing a minimum of three active electrodes were included. Neuronal firing metrics were exported as the averages from each well from Axion Biosystems’ Neural Metrics Tool.

### Seahorse assay using iPSC-derived excitatory neurons

For bioenergetic profile in iPSC-derived excitatory neurons, seahorse assay was performed using the Seahorse XF Cell Mito Stress Test kit and the Seahorse XF Glycolysis Stress kit according to the manufacturer’s instructions (Agilent) as previously described (*59*). Briefly, for mitochondrial respiration, iPSC-derived excitatory neurons were differentiated as described in a Seahorse XF96 cell culture microplate. At DIV20 of differentiation, the medium was replaced with 180 μl of media for the mitochondrial respiration stress test (base medium with 2 mM l-glutamine, 1 mM sodium pyruvate, and 10 mM glucose). Before the experiment, the cells were incubated for 1 hour at 37°C in a non-CO_2_ incubator. Changes in oxygen consumption were measured following treatment with oligomycin (1.5 μM), carbonyl cyanide *p*-trifluoromethoxyphenylhydrazone (1 μM), and rotenone (0.5 μM) plus antimycin A (0.5 μM). The OCR measured three times before treatment and after every injection. For glycolytic function, iPSC-derived excitatory neurons were differentiated as described in a Seahorse XF96 cell culture microplate. At DIV20 of differentiation, the medium was replaced with 180 μl of media for the glycolysis stress test (base medium with 2 mM l-glutamine). Before the experiment, the cells were incubated for 1 hour at 37°C in a non-CO_2_ incubator. Changes in oxygen consumption were measured following treatment with glucose (10 mM), oligomycin (1.5 μM), and 2-deoxyglucose (1 μM). The ECAR measured three times before treatment and after every injection. Each data point was normalized to the first data point to calculate the OCR or ECAR ratio. Data were processed and analyzed using the Seahorse Wave software.

### Human postmortem brain sample preparation

This study was conducted in accordance with a protocol approved by the Mayo Clinic Institutional Review Board (no. 15-009452). Autopsy-confirmed control and LBD brains were obtained from the brain bank for neurodegenerative disorders at Mayo Clinic in Jacksonville, including 3 control and 5 LBD cases (including one *SNCA* duplication and one triplication case) for snRNA-seq (table S4), 11 control and 11 LBD cases for qPCR validation (table S7), and 16 control and 16 LBD cases for Western blotting validation (table S8). All cases had standardized neuropathologic sampling and evaluation as described previously (*6*, *60*, *61*). Briefly, thioflavin-S fluorescence microscopy was used to evaluate AD neuropathologic change, including Braak neurofibrillary tangle stage and Thal amyloid phase (*62*, *63*). Lewy-related pathology was assessed with α-SYN immunohistochemistry (in house, 1:3000) and Lewy bodies were counted in a medium power field (20×) in the cingulate gyrus, inferior parietal lobule, middle frontal gyrus, parahippocampal gyrus, and superior temporal gyrus, as well as the amygdala. Lewy body pathology was also documented in basal nucleus of Meynert, substantia nigra, locus ceruleus and dorsal motor nucleus of the vagus, as well as olfactory bulb, if available (*6*, *64*). The sample characteristics of these cohorts are summarized in tables S4, S7, and S8.

### Single-nucleus isolation of human brains for RNA library preparation

Nuclei were isolated from the frozen superior temporal cortices as described previously with some modifications (*65*). Specifically, the frozen tissue samples weighing 50 to 60 mg were briefly exposed to 2 ml of Nuclei EZ Lysis Buffer (Sigma-Aldrich), supplemented with EDTA-free protease inhibitors (Sigma-Aldrich) and recombinant ribonuclease (RNase) inhibitors (Takara Bio USA) at 4°C. The tissue was then homogenized using a glass Dounce tissue grinder through 10 to 15 strokes with pestle A, followed by 10 strokes with pestle B. Then, an additional 2 ml of lysis buffer containing protease and RNase inhibitors was added to the homogenized tissue, followed by 5-min incubation. Subsequently, the nucleus suspension was filtered using a 40-μm cell strainer and pelleted by centrifugation at 300*g* for 5 min. The nucleus pellets were resuspended in 900 μl of debris removal solution (Miltenyi Biotech) containing RNase inhibitors (0.4 U μl^−1^) and 3.1 ml of calcium and magnesium-free Dulbecco’s PBS (DPBS). Next, 4 ml of DPBS was carefully overlayed onto the nuclei resuspension was performed, followed by another centrifugation at 3000*g* for 5 min. After centrifugation, the supernatant was discarded, and the nuclei were resuspended in the solution consisting of 1% bovine serum albumin in DPBS, supplemented with RNase inhibitors (0.4 U μl^−1^). Throughout the process, the nuclei were kept on ice or at 4°C. Last, the density of the nuclei suspension was calculated using a C-chip–disposable hemocytometer (INCYTO) with trypan blue staining (Bio-Rad). RNA library preparation was performed on isolated organoid samples using the chromium platform (10x Genomics) with the Chromium Next GEM Single Cell 3′ v3.1 kit, using a targeted input of 5000 cells per sample as described above.

### snRNA-seq data analysis

The snRNA-Seq data were processed and quantified using the 10x Genomics Cell Ranger Software Suite (version 3.1.0) with a customized “pre-mRNA” human reference (GRCh38-1.2.0.pre-mrna), which was constructed as per the procedure outlined by 10x Genomics (https://support.10xgenomics.com/single-cell-gene-expression/software/pipelines/latest/advanced/references). Subsequently, each individual’s gene count matrix was imported into the Seurat package (version 3.1.0) for subsequent analysis. Cells with gene count below 200 or mitochondrial genes over 5% were excluded from the downstream analysis (*65*–*67*). Genes expressed by less than five cells were also excluded. For each sample, the distribution of the gene number, UMI, and mitochondrial percentage, as well as their interrelations, were meticulously examined to identify outlier cells or samples with excessively low gene counts, unique molecule counts, or high mitochondrial gene percentages for exclusion. The ambient RNA contamination of the nuclei was estimated using the decontX function from the celda R package (v.1.10.0) (*68*), and the doublets identified using the DoubletFinder R package (v.2.0.3) were removed from the downstream analysis (*69*).

#### Cell clustering and annotation

Cell clustering analysis was conducted using the Harmony package (v.1.0) implemented in the Seurat package (v.4.3.0) following the standardized workflow (*70*). The top 3000 highly variable genes identified through variance-stabilizing transformation were used for the downstream analysis. nFeature_RNA, percent.mt, and decontX_contamination were regressed out for data scaling, and the top 50 PCs were chosen for cell clustering using the RunHarmony function, with each sample treated as one batch. The cell clusters were annotated on the basis of their expression of known marker genes. To maximize the fidelity of cluster annotation, we removed subclusters showing high expression of marker genes belonging to different cell types. The identity of each cell cluster was further confirmed using their enriched genes identified through differential gene expression (DEG) with the MAST package (v1.20.0) (*71*). Specifically, we compared the gene expression of each cluster versus all other clusters combined using the two-part hurdle models adjusted for the gene detection rate, percent.mt, and decontX_contamination.

#### Differential gene expression analysis

The differential gene expression analysis for the major cell types, including excitatory neurons, inhibitory neurons, astrocytes, oligodendrocytes, OPCs, microglia, and vascular cells, were performed by fitting two-part hurdle models with the sample ID as the random effect using the MAST package (v1.20.0). All models were adjusted for gene detection rate, age, and percent.mt, decontX_contamination, sequencing saturation, and the RIN number of the sample. Mitochondrial genes, antisense, divergent, and overlapping transcripts, and genes with zero count in more than 90% of the cells in each major or subcluster of cells were excluded from the DEG analysis. The *P* values were corrected by FDR adjustment. Genes with absolute log_2_FC equal or greater than 0.1 and FDR less than 0.05 were defined as DEGs. The DEGs were used as inputs for IPA.

### RNA extraction from organoid and human brain for qPCR analysis

Total RNA was isolated from organoids using a NucleoSpin RNA Mini kit (Macherey-Nagel) and from frozen superior temporal cortices of postmortem human brains using TRIzol reagent (Qiagen) and rNeasy Mini Kit (Qiagen). The RNA was subjected to deoxyribonuclease I digestion to remove contaminating genomic DNA. Reverse transcription of RNA was performed using iScript Reverse Transcription Supermix (Bio-Rad). cDNA was added to a reaction mix containing gene-specific primers and SYBR Green Supermix (Bio-Rad). All samples were run in duplicate and were analyzed with QuantStudio Real-Time PCR Software (Life Technologies). The relative gene expression was normalized to *GAPDH* expression. Primer sequences and information are as follows: *GAPDH*, Hs.PT.39a.22214836; *FADS1*, Hs.PT.58.40683791; *PRKN*, Hs.PT.58.40583803; *ABCB10*, Hs.PT.58.39325464.

### Protein extraction from human brain

Frozen superior temporal cortices were subjected to sequential protein extractions using TBS buffer and detergent-containing buffer (TBSX) to obtain the buffer-soluble (TBS) and detergent-soluble (TBSX) proteins, respectively. Briefly, cortices were homogenized in ice-cold TBS buffer containing a protease inhibitor cocktail (Roche) and a phosphatase inhibitor (Roche), sonicated, and incubated at 4°C for 30 min with end-over-end agitation. The supernatant was collected after centrifugation at 100,000*g* for 1 hour at 4°C as TBS fraction. The residual pellet was rehomogenized in TBSX buffer with protease and phosphatase inhibitors, sonicated, incubated at 4°C for 30 min with end-over-end agitation, and centrifuged as above to obtain supernatant as the TBSX fraction for Western blot.

### Drug screening by α-SYN RT-QuIC assay

To identify inhibitors of α-SYN seeding and aggregation, we conducted the RT-QuIC assay using brain lysates obtained from a cohort of human brains with both sever AD type and Lewy pathologies. The brain lysates were pooled from five cases as used in our previous publication (*22*). RT-QuIC assay was performed as previously described in a 96-well clear-bottom plate with minor modifications for drug treatment (*22*). The reaction mixture consisted of final concentrations of 40 mM phosphate buffer (pH 8.0), 170 mM NaCl, 10 μM thioflavin T (ThT), 0.0006% SDS, and recombinant α-SYN (0.1 mg/ml; Proteos). To seed the RT-QuIC reactions, the TBS brain lysates were mixed with α-SYN RT-QuIC reaction mixture per well of a 96-well plate preloaded with six 0.8-mm silica beads (OPS Diagnostics). Each drug from a 1280–FDA-approved drug library (Prestwick Chemical) was treated at different concentrations (as indicated in Fig. 5, A to E) in each duplicated well as well as DMSO and EGCG for negative and positive control, respectively. Screenings were conducted using 16 of the 96-well plates for primary analysis, 4 of the 96-well plates for secondary analysis, and a single 96-well plate for tertiary analysis to minimize batch effects. DMSO and EGCG were included as negative and positive controls, respectively, in each 96-well plate. Next, plates were sealed with a plate sealer (Nalgene Nunc International), and reactions were initiated in a FLUOstar Omega plate reader (BMG LABTECH Inc.) with alternating 1-min shake and rest cycles (double orbital, 400 rpm) at 40°C. ThT fluorescence readings were recorded at excitation and emission wavelengths of 450 and 480 nm, respectively, every 30 min over a period of 60 hours. Drug efficiency was calculated by comparing the maximal fluorescence levels around the 30-hour time point in FDA-approved drug-treated wells to those in negative control wells within each 96-well plate.

### Transmission EM analysis

To observe morphology of α-SYN aggregates, the aggregated end products of the RT-QuIC reaction with treatment of each drug at 0.5 μM indicated in fig. S10 as well as DMSO and EGCG as control were centrifuged at 20,000*g* for 30 min at 4°C. After discarding the supernatants, pellets were suspended with 20 μl of PBS for negative stain transmission EM. Samples (4 μl) were deposited onto 400-mesh carbon-coated grids (Agar Scientific) and incubated for 1 min before blotting the excess solution off. Grids were washed with water and blotted dry before negatively staining the samples with 4 μl of filtered 0.5% uranyl acetate for 1 min. Grids were then dried with filter paper and left to air dry for 5 min before storage. Electron micrographs were obtained using a Jeol JEM-1400Flash transmission electron microscope (Jeol) fitted with a Matataki 4 M Flash camera Gatan camera at an operating voltage of 80 kV as previously described (*72*).

### Computational drug-working modeling

#### Computational tools

All the modeling studies were conducted using commercial molecular modeling package Schrodinger Maestro (Schrödinger Release 2023-2: Schrödinger, LLC, New York, NY, 2023) and PyMOL 2.5 (The PyMOL Molecular Graphics System, Version 2.5, Schrödinger, LLC). The computational work was performed on Exxact Valence Deep Learning & AI Workstation with NVIDIA RTX A5000 X2 (20,000 GPUs) + A2000 graphics card with 32 processors running on Rocky Linux operating system.

#### α*-SYN aggregates target preparation*

The Protein Preparation Wizard module in Schrodinger Maestro was used to prepare the structure of α-SYN aggregates using the Protein Data Bank identification code: 6L1T (*73*). In the preparation process, the proteins within the α-SYN structure (6L1T) underwent automated adjustments, including removal of structural bumps; correction of bond order, bond length, and torsion angle; addition of hydrogens; correction of charges; and filling of missing residues using the workspace analyzer. The structure was then subjected to a minimization process. Our protocols for relaxation and docking used established and recognized procedures (*74*–*80*).

#### Grid generation

The optimized structure of the α-SYN aggregate was used to construct a lattice box using the Glide Grid module. As the specific binding site of the active compounds within the α-SYN aggregate was unknown, a blind docking approach was used to identify potential binding sites. Three sets of docking grid sites were determined for the inhibitors used in the study. Three independent grid boxes were created for each docking study, with the following grid center points: (X: 146.66, Y: 206.43, Z: 205.06), (X: 179.74, Y: 205.24, Z: 201.75), and (X: 159.32, Y: 199.13, Z: 206.04). The box size for all three grid boxes was set at 76 Å by 76 Å by 76 Å.

#### Ligand preparation

Before docking with α-SYN aggregate targeted molecules, the active compounds D1, D3, D4, D6, and negative controls mepenzolate and metixene were prepared using the LigPrep module in Maestro. The Optimized Potentials for Liquid Simulations 2005 (OPLS2005) force field was used to construct and refine the topology of the compounds for an entirely aqueous environment. To reduce the geometric complexity, conformational space, tautomers, ionization states, and stereoisomers were explored for each compound. The LigPrep module facilitated the generation of 3D structures from their corresponding 2D representations.

#### Molecular docking

In ligand docking, the identification of inhibitor binding sites on α-SYN protein assembly is crucial as it reveals the interaction sites between the inhibitors and the amino acid residues of the target α-SYN aggregates. It also provides insights into how the inhibitors structurally and dynamically interfere with the native pathogenic state of the aggregates. As the druggable site of α-SYN aggregates was unknown for this study, we used overlapping grid boxes that covered the entire protein surface to explore the complete conformational space during exhaustive docking. To locate the inhibitor binding region, blind docking was used to examine potential binding sites on the protein molecule (α-SYN). While each drug molecule exhibited numerous potential binding sites, we filtered them on the basis of their site score and volume filling capacity for the pocket or cavity. In this in silico approach, we assessed the compatibility of each binding mode for compounds D1, D3, D4, and D6 with the α-SYN protein and their implications on the assembly of the stacked tetramer that forms a head-to-head dimer. Through our analysis, the tested drug compounds revealed promising putative drug sites based on favorable docking metrics obtained from the XP docking scoring function of Glide (Schrödinger Release 2023-2: Schrödinger, LLC, New York, NY, 2023). Docking triplicates were performed for each inhibitor’s binding mode to validate and establish the docking criteria, enabling further analysis and interpretation of the results.

### Statistical analysis

All data were reported as mean values ± SEM unless elsewise indicated. With the sample size ≤8, Student’s *t* test was used to compare outcomes among groups. For MEA assay, two-way repeated measures analysis of variance (ANOVA) was used to compare outcomes among groups. Statistical analyses were performed using Excel or GraphPad Prism v8.4.3 (GraphPad Software).
