# Supplementary material for: Modeling Lewy body disease with SNCA triplication iPSC-derived cortical organoids and identifying therapeutic drugs
Source: Sci Adv. 2024 Sep 11;10(37):eadk3700. doi: 10.1126/sciadv.adk3700 (PMC11389790; doi:10.1126/sciadv.adk3700)
Supplement: Supplementary file 1 — Figs. S1 to S12 Tables S1, S4, S7 and S8 Legends for tables S2, S3, S5, S6, S9 and S10 [file sciadv.adk3700_sm.pdf]

Science Advances
AAAS

Supplementary Materials for

Modeling Lewy body disease with *SNCA* triplication iPSC-derived cortical organoids and identifying therapeutic drugs

Yunjung Jin *et al.*

Corresponding author: Na Zhao, zhao.na@mayo.edu

Sci. Adv. **10**, eadk3700 (2024)
DOI: 10.1126/sciadv.adk3700

The PDF file includes:

Figs. S1 to S12
Tables S1, S4, S7 and S8
Legends for tables S2, S3, S5, S6, S9 and S10

Other Supplementary Material for this manuscript includes the following:

Tables S2, S3, S5, S6, S9 and S10

Supplementary Figures

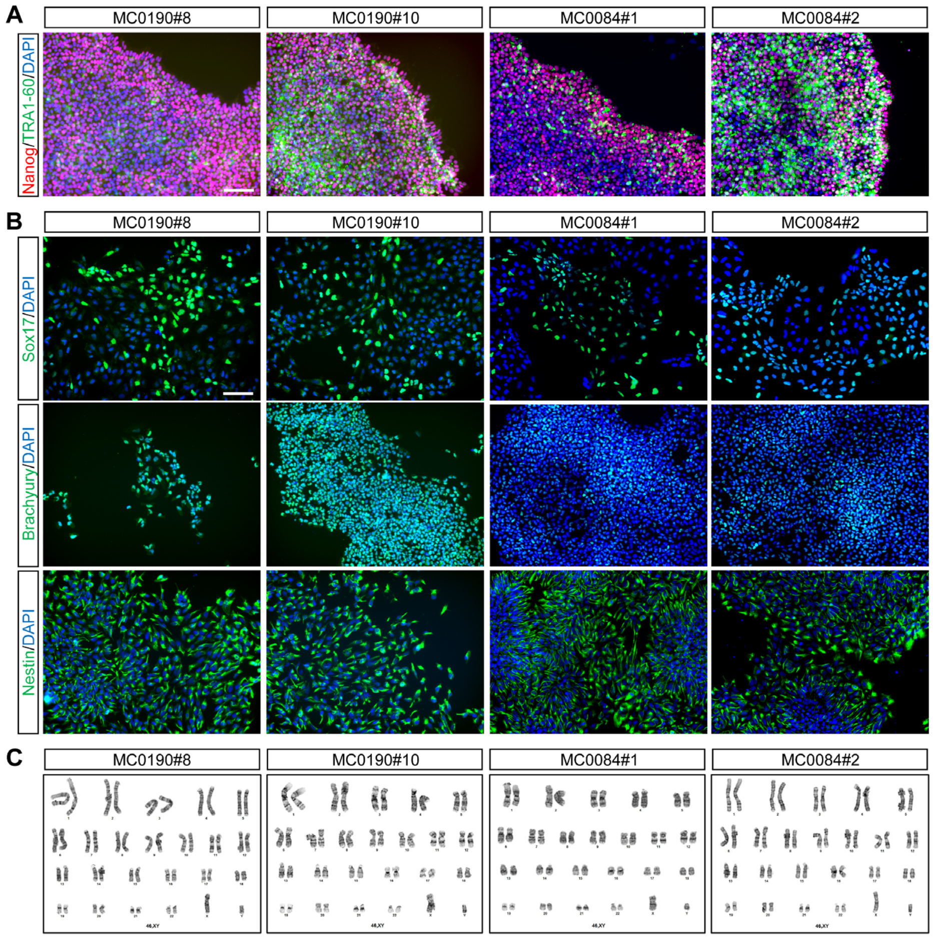

Fig. S1. Characterization of *SNCA* triplication iPSCs

Two independent subclones derived from two *SNCA* triplication (SNCA Tri) iPSC lines were used and characterized for cortical organoid differentiation.

(A) Immunofluorescence staining of pluripotency markers, Nanog (red) and TRA1-60 (green), in the iPSC lines and subclones. Nuclei were stained with DAPI (blue). Scale bar, 100 μm.

(B) Differentiation of iPSC lines into three germ-layer cells. Immunofluorescence staining for endodermal marker (Sox17, green), mesodermal marker (Brachyury, green), and ectodermal marker (Nestin, green). Nuclei were stained with DAPI (blue). Scale bar, 100 μm.

(C) Karyotyping analysis of the iPSC lines and subclones.

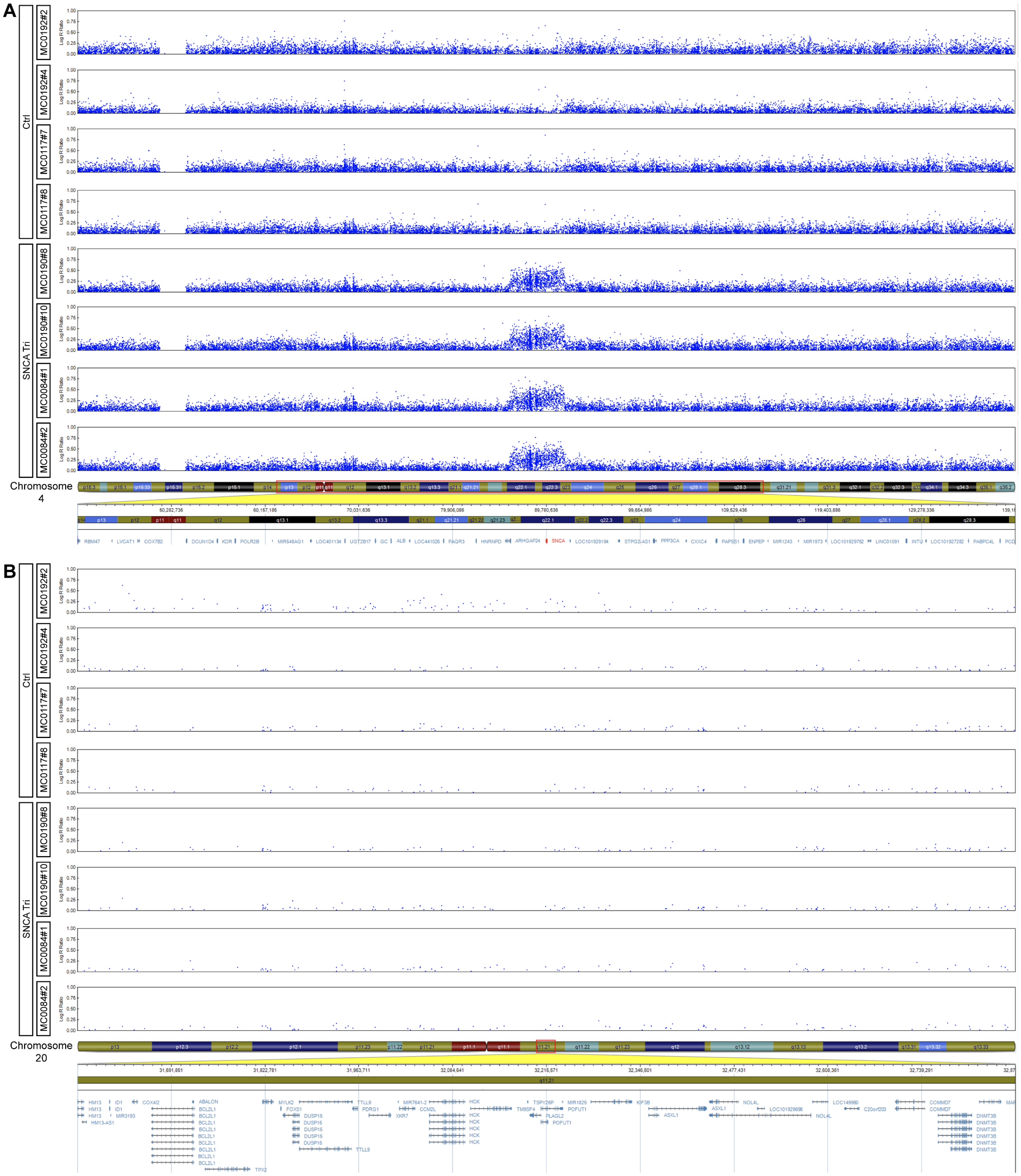

Fig. S2. Copy number variations (CNV) in control and *SNCA* triplication iPSCs

(**A-B**) Two independent subclones derived from two control (Ctrl) and *SNCA* triplication (SNCA Tri) iPSC lines were used to identify CNV in *SNCA* gene-coding regions in chromosome 4 (**A**), and CNV hotspot regions in iPSCs, including *BCL2L1*, *ID1* and *DNMT3B* genes, in chromosome 20 (**B**).

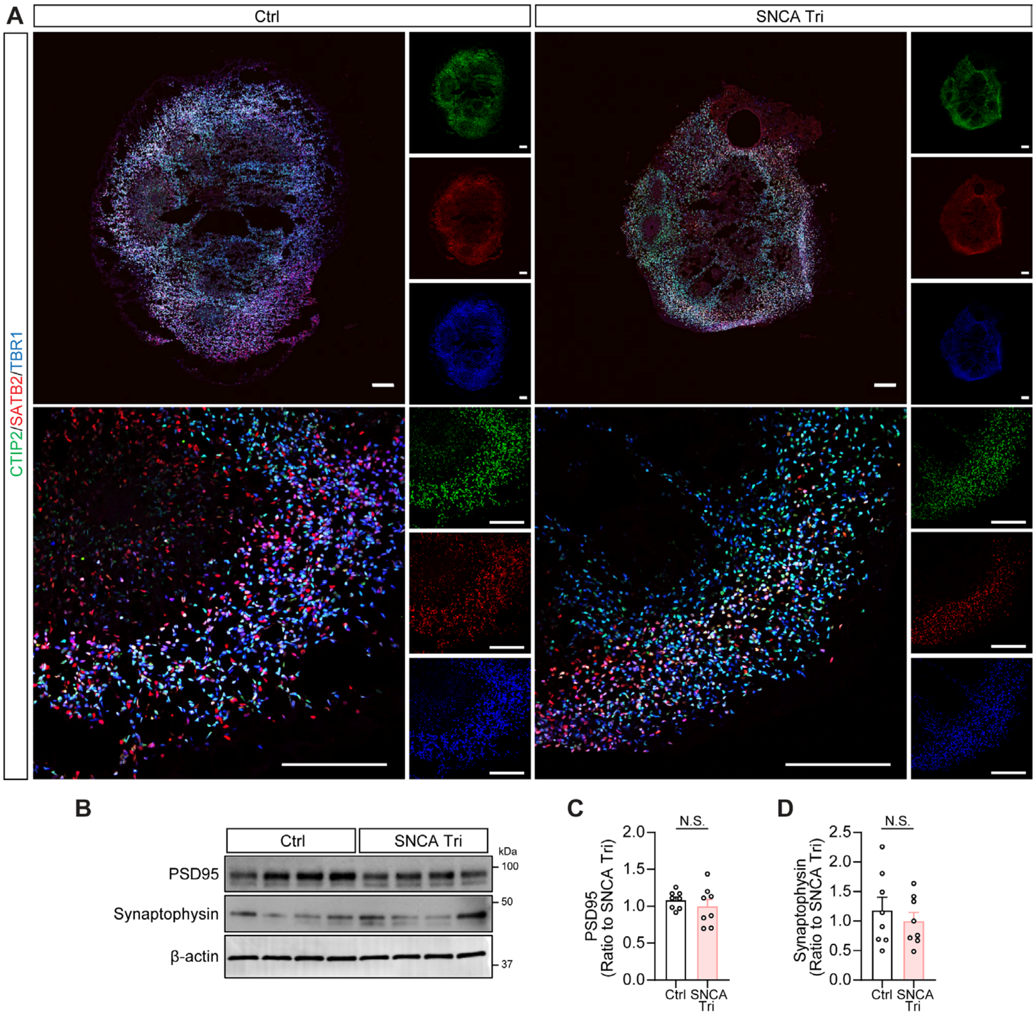

Fig. S3. Characterization of control and *SNCA* triplication organoids

(A) The Ctrl and SNCA Tri organoids were immunostained with cortical layer markers, CTIP2 (green), SATB2 (red) and TBR1 (blue), with lower magnification images (upper panel) and higher magnification images (lower panel). Scale bar, 200 μm.

(B-D) Representative gel images and quantification of PSD95 and Synaptophysin levels, as detected by western blotting using lysates from TBSX fractions of Ctrl and SNCA Tri organoids are presented. Results were normalized to β-actin levels. N = 4 samples with two replicates per group. Each dot on the graph represents an individual replicate. Molecular weight markers (kDa) are indicated on the right side of each blot.

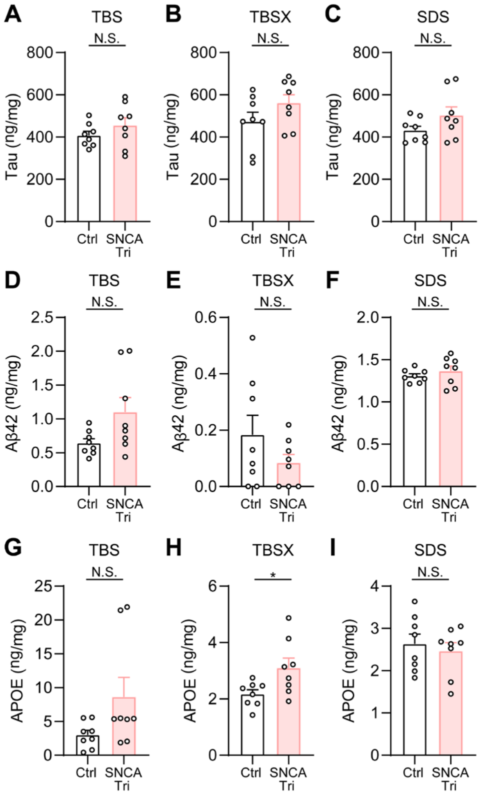

Fig. S4. Levels of Tau, Amyloid-β 42 (Aβ42), and APOE in cortical organoids

The levels of total Tau (**A-C**), Aβ42 (**D-F**), and APOE (**G-I**) in the TBS, TBSX, and SDS fractions were measured by ELISA and compared between Ctrl and SNCA tri organoids. Experiments were carried out in duplicate. Data represent mean ± SEM. N = 4 samples per group, each dot represents an individual replicate.

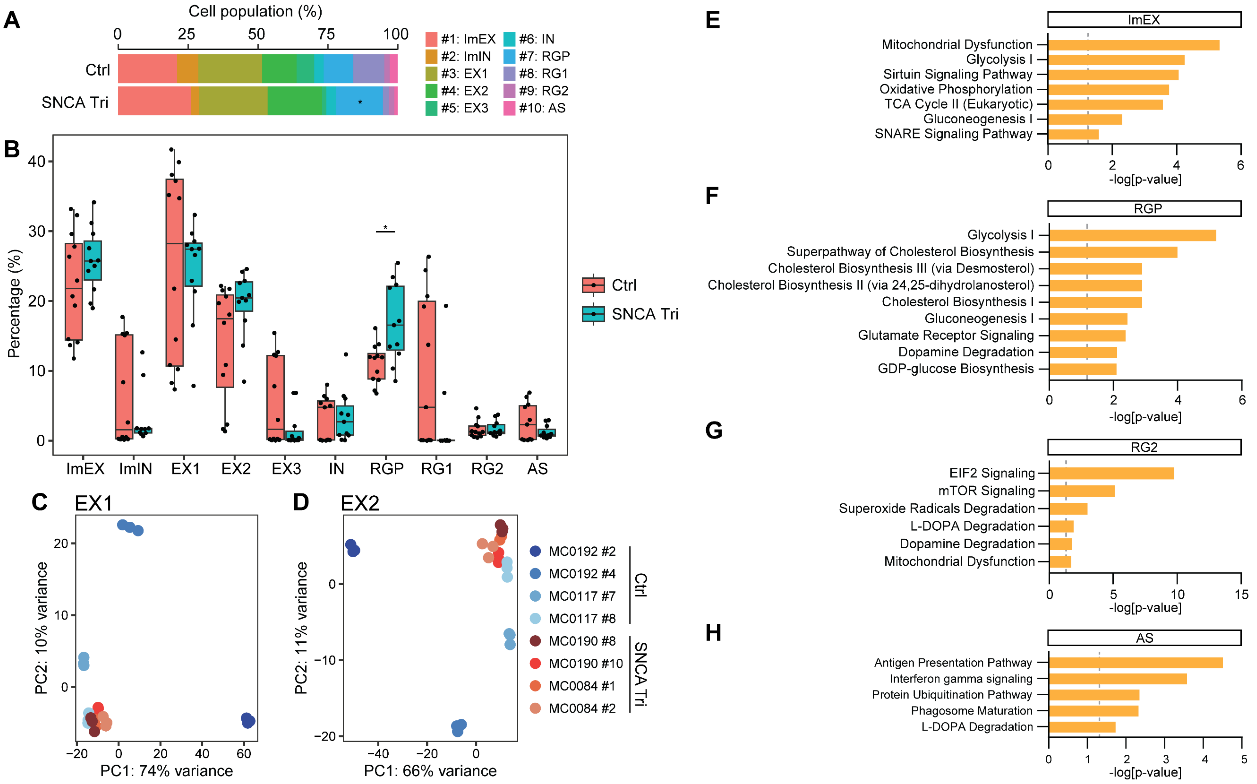

Fig. S5. Cell composition, sample variation, and pathway enrichment in cortical organoids from scRNA-seq data

(A and B) Bar graph (**A**) and box plot (**B**) showing the proportion of each cell types of Ctrl and SNCA Tri organoids. #1: Immature excitatory neuron (ImEX); #2: Immature inhibitory neuron (ImIN); #3: Excitatory neuron 1 (EX1); #4: Excitatory neuron 2 (EX2); #5: Excitatory neuron 3 (EX3); #6: Inhibitory neuron (IN); #7: Radial glia progenitor (RGP); #8: Radial glia 1 (RG1); #9: Radial glia 2 (RG2); #10: Astrocyte (AS). Statistical analyses were conducted using Wilcoxon rank-sum test. $*p < 0.05$

(C and D) Principal component analysis (PCA) plot showing the variation of the organoids derived from different lines and subclones in EX1 (**C**) and EX2 (**D**) clusters.

(E-H) Selected canonical pathways enriched by DEGs from the ImEX (**E**), RGP (**F**), RG2 (**G**), and AS (**H**) clusters.

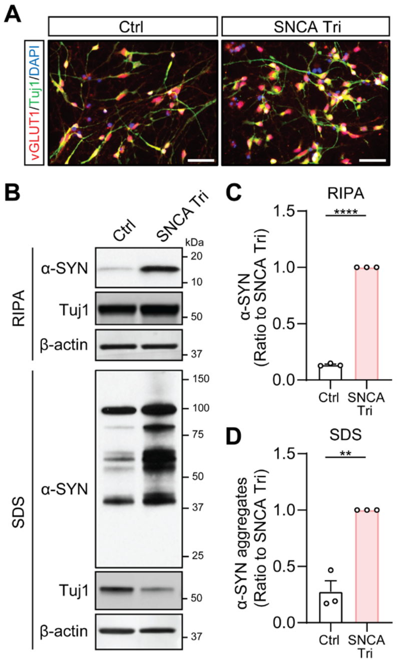

Fig. S6. Characteristics of iPSC-derived excitatory neurons

(A) Immunofluorescence staining of Ctrl and SNCA Tri neurons at DIV15 with excitatory neuronal markers, vGLUT1 (red) and Tuj1 (green), and nuclear stain DAPI (blue). Scale bar, 50 μm.

(B-D) Western blot analysis of Ctrl and SNCA Tri neurons at DIV20. Neuronal lysates were sequentially fractionated into soluble fraction (RIPA) and insoluble fraction (SDS). The levels of total α-SYN in the RIPA (**B and C**) and SDS (**B and D**) fractions were measured and compared between Ctrl and SNCA Tri neurons. Results were normalized to Tuj1 levels. Molecular weight markers (kDa) are shown on the right side of each blot. Three independent experiments were performed. Statistical analyses were conducted using Student t-tests. $^{**}p < 0.01$; $^{****}p < 0.0001$.

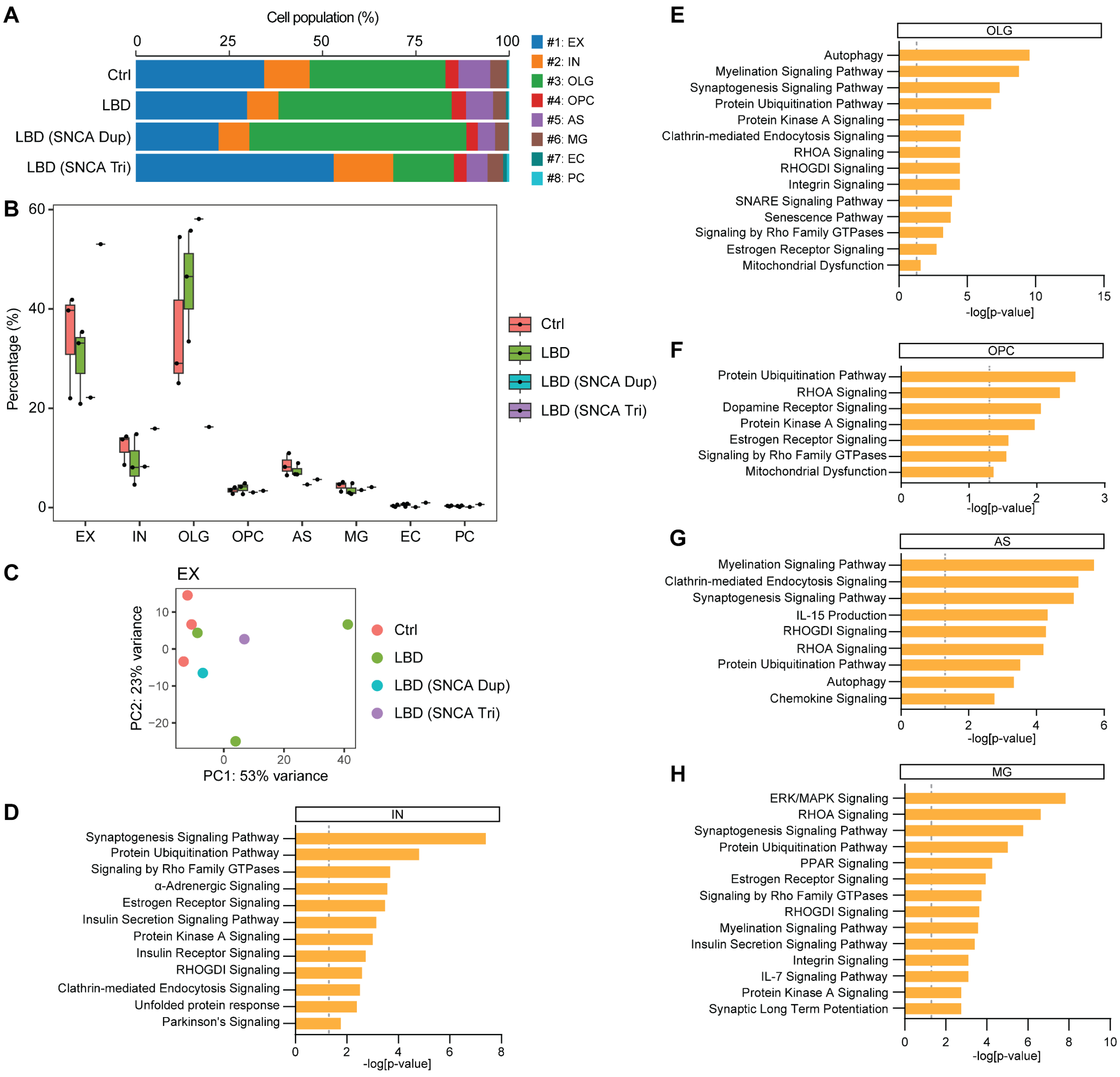

Fig. S7. Cell composition, sample variation, and pathway enrichment in postmortem human brains from snRNA-seq data

(A and B) Bar graph (**A**) and box plot (**B**) showing the proportion of each cell type in Ctrl, LBD, LBD (SNCA Dup), and LBD (SNCA Tri) brains. #1: Excitatory neuron (EX); #2: Inhibitory neuron (IN); #3: Oligodendrocyte (OLG); #4: Oligodendrocyte progenitor cell (OPC); #5: Astrocyte (AS); #6: Microglia (MG); #7: Endothelial cell (Endo); #8: Pericyte (Peri).

(C) PCA plot showing the sample variation in EX cluster.

(D-H) Selected canonical pathways enriched by DEGs from IN (**D**), OLG (**E**), OPC (**F**), AS (**G**), and MG (**H**) clusters.

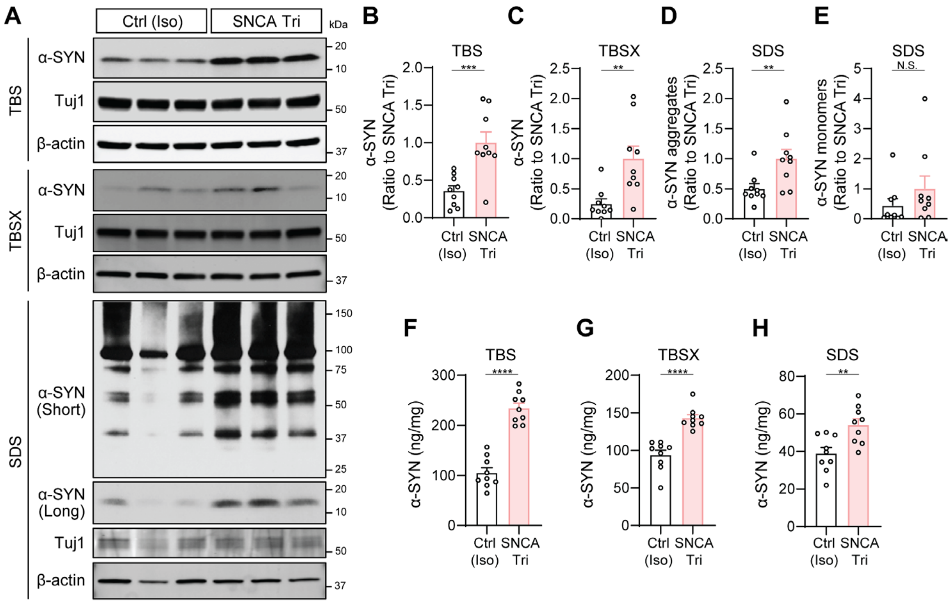

Fig. S8. Increased levels of α-SYN in the *SNCA* triplication and isogenic control cortical organoids

IPSC lines from *SNCA* triplication (SNCA Tri) and isogenic control (Ctrl (Iso)) were differentiated into cortical organoids, and harvested after 2 months of differentiation.

(A-E) The levels of total α-SYN in TBS, TBSX, and SDS fractions were measured using western blotting and compared between Ctrl (Iso) and SNCA Tri organoids. Different exposure times (Short and Long) were applied to display and quantify the aggregated and monomeric α-SYN. Results were normalized to Tuj1 levels. Molecular weight markers (kDa) are indicated on the right side of each blot.

(F-H) The levels of total α-SYN in the TBS, TBSX, and SDS fractions were measured using ELISA and compared between the Ctrl (Iso) and SNCA Tri organoids.

The experiments were conducted in triplicate with three independent experiments. Each dot on the graph represents an individual replicate. Data represent mean ± SEM. Student t-tests were used for statistical analyses. *$p < 0.05$; **$p < 0.01$; ***$p < 0.001$; ****$p < 0.0001$; N.S., not significant.

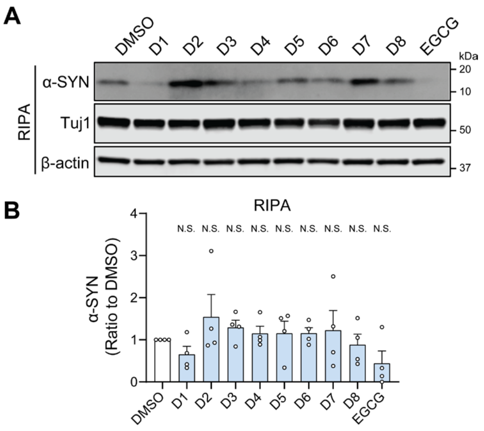

Fig. S9. Effects of drug treatment on soluble α-SYN levels in *SNCA* triplication organoids

(A and B) SNCA Tri organoids were treated with the 8 drug candidates at 0.5 μM for two weeks. The organoids were lysed in RIPA and SDS buffers sequentially, and the levels of total α-SYN in the RIPA were measured by western blot. DMSO and EGCG were used as controls. Four independent experiments were conducted, and data are presented as mean ± SEM. Student t-tests were used for statistical analyses when comparing each treatment group to DMSO controls. N.S., not significant.

A

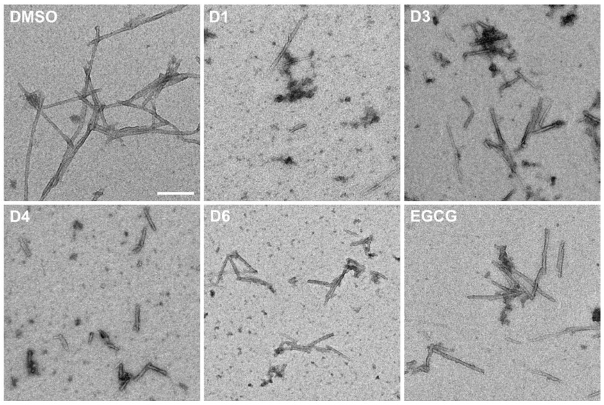

Fig. S10. Electron microscopy (EM) analysis of α-SYN fibrilization

(A) Representative electron microscopy (EM) images of α-SYN-RT-QuIC products derived from TBS brain lysates with 5 μM drug treatment (D1, D3, D4, and D6) are displayed. Scale bar, 200 nm.

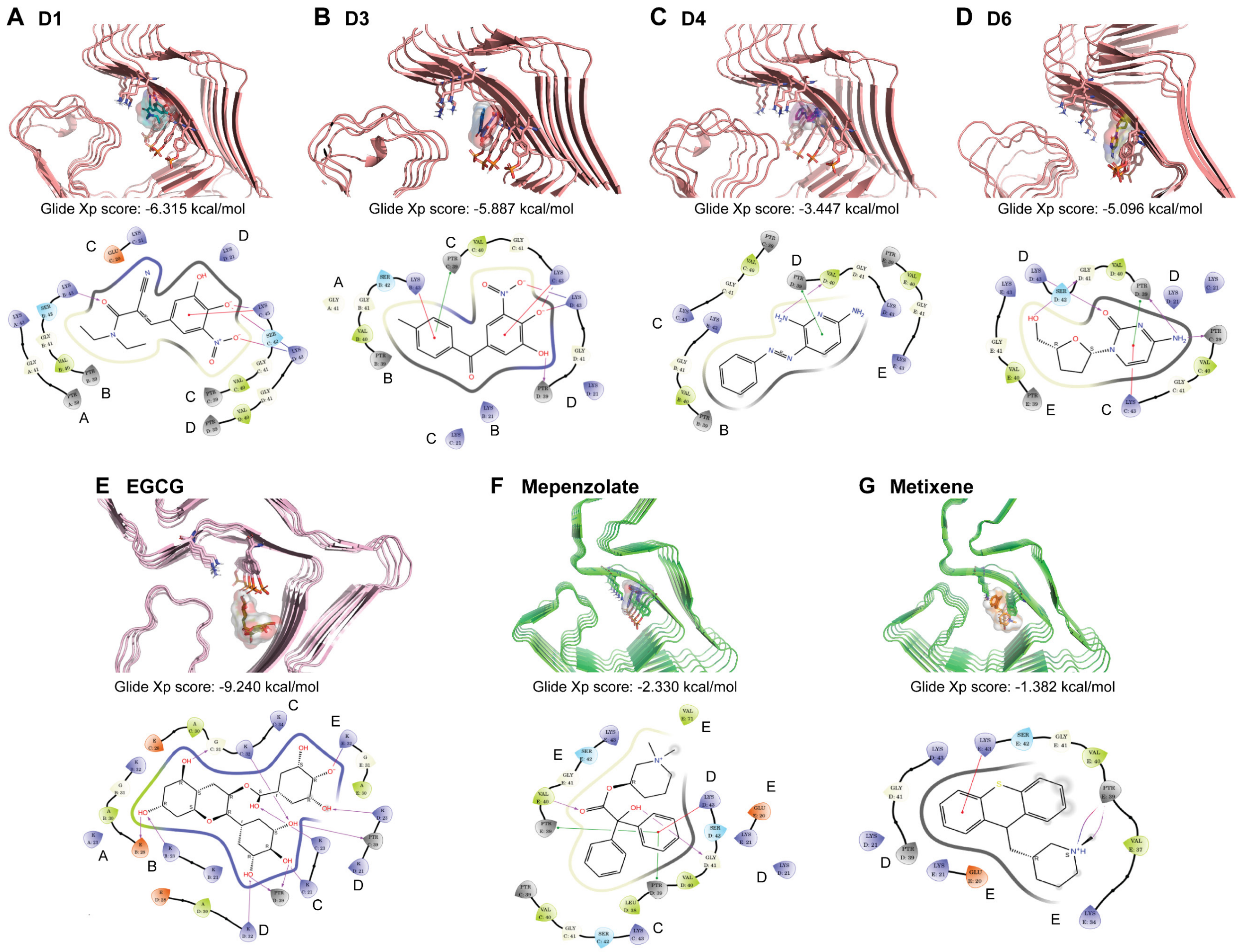

Fig. S11. Computational mapping of drug interactions with α-SYN aggregates

The 3D computational structure and binding mode of the four active compounds (A: D1, B: D3, C: D4, D: D6) and negative drugs (E: Mepenzolate, F: Metixene) in α-SYN aggregates are shown (upper panel). Additionally, the drug molecules are represented in flat 2D format, with amino acid teardrops indicating the direction of the sidechain (pointing away or towards the drugs, lower panel). Interactions, such as π-π stacking, hydrogen bonding, van der Waals (VdW) interactions, or solvent effects, are illustrated.

(A) D1 binding mode. D1 (Glide Xp score: -6.315 kcal/mol) engages in hydrophobic contacts with phosphorylated Tyr39, Gly41, Lys43 in the A, B, C, D chains of α-SYN aggregates, as well as Val40 of the B, C, D chains, Ser42 of the B chain, and Glu20 of the C chain. The nitro group of D1 forms an electrostatic interaction/salt bridge with Lys43 of the D chain, while the deprotonated hydroxyl moiety stabilizes the complex. Furthermore, a π-cation stacking occurs between D1's phenyl ring and Lys43 of the C chain,

reinforcing the interaction. The hydroxyl moiety of the inhibitor also forms a hydrogen bond with Lys43 of B chain.

(B) D3 binding mode. D3 (Glide Xp score: -5.887 kcal/mol)'s phenyl ring forms π-cation stacking with Lys43 of the B and C chains in α-SYN aggregates. The nitro and deprotonated hydroxyl groups of D3 establish salt bridges with Lys43 of the C and D chains, respectively. Additionally, the hydroxyl moiety of D3 forms a hydrogen bond with phosphorylated Tyr39 of the D chain. D3 establishes numerous hydrophobic contacts with phosphorylated Tyr39, Lys21, Lys43 in the B, C, and D chains, as well as Val40 in the B, C, D chains, Ser42 in the B and C chains, and Gly41 from the A and B chains. A stable π-π stacking interaction is observed between the D3's phenyl ring and phosphorylated Tyr39 of the C chain.

(C) D4 binding mode. D4 (Glide Xp score: -3.447 kcal/mol) forms a hydrogen bond between its amine group and Val40 of the D chain in α-SYN aggregates, and a π-π stacking between its pyridine ring and phosphorylated Tyr39 of the D chain. Additionally, D4 engages a series of hydrophobic contacts with phosphorylated Tyr39, Val40, Gly41 and Lys43 of the B, C, D, E chains, respectively.

(D) D6 binding mode. D6 (Glide Xp score: -5.096 kcal/mol) has a π-π stacking between its pyrazine ring and phosphorylated Tyr39 of the D chain, as well as a π-cation interaction stacking. The NH2 group of D6 forms a hydrogen bond with phosphorylated Tyr39 of the C and D chains. Similarly, the hydroxyl oxygen of D6 forms a hydrogen bond with Gly41 of the D chain, and Lys43 of the D chain forms a hydrogen bond with the oxygen attached to the pyrazine ring. D6 also engages in additional hydrophobic interactions with Lys43, phosphorylated Tyr39, Val40, Gly41, and Lys43 of the C, D, E chains of SYN aggregates, as well as hydrophobic interaction with Ser42 of the D chain.

(E) EGCG binding mode. EGCG (Glide Xp score: -9.240 kcal/mol) binds to several hydrogen bonds with Lys residues due to the presence of hydroxy moieties. The complex was also stabilized by few hydrophobic interactions.

(F and G) Negative controls Mepenzolate (**F**) and Metixene (**G**) do not induce any structure changes in α-SYN aggregates, as indicated by their poor Xp docking scores (-2.330 kcal/mol and -1.382 kcal/mol, respectively).

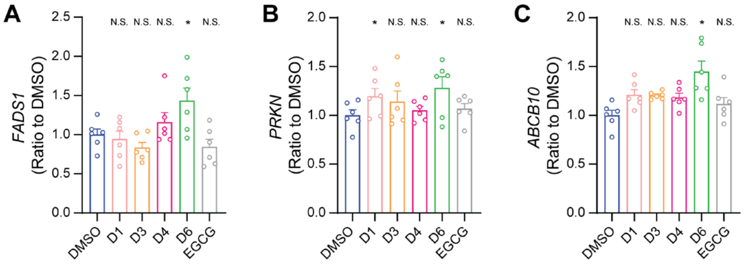

Fig. S12. Effects of the drug treatment on *FADS1*, *PRKN*, and *ABCB10* gene expression in *SNCA* triplication iPSC-derived cortical organoids

SNCA Tri organoids were treated with the four drug candidates (D1, D3, D4, and D6) at 0.5 μM for two weeks. RNAs were extracted and qPCR was performed to analyze the RNA levels of *FADS1* (**A**), *PRKN* (**B**) and *ABCB10* (**C**) in organoids. Six independent experiments were conducted, and data are presented as mean ± SEM. Student t-tests were used for statistical analyses by comparing each group with DMSO-treated group. *$p < 0.05$; N.S., not significant.

Supplementary Tables

Table S1. The information of human iPSC lines

	Line ID	Colony ID	Age	*Sex*	*SNCA* allele number	Source
**Ctrl**	MC0192	#2 and #4	83	F	2	Fibroblast
**Ctrl**	MC0117	#7 and #8	71	M	2	Fibroblast
**SNCA Tri**	MC0190	#8 and #10	25	M	4	Fibroblast
**SNCA Tri**	MC0084	#1 and #2	26	M	4	Fibroblast
**SNCA Tri**	SNCA3x_0KO	C1	42	M	4	Commercial ATCC
**Ctrl (Isogenic)**	SNCA3x_2KO	C1	42	M	2	Commercial ATCC

Table S4. Patient characteristics of the postmortem human brains for snRNA-seq

	Age	Sex	*SNCA* allele number	Braak stage	Thal phase
**Ctrl**	68	M	2	1	0
**Ctrl**	67	M	2	1	0
**Ctrl**	59	M	2	0	0
**LBD**	68	M	2	2	1
**LBD**	55	M	2	3	0
**LBD**	66	M	2	3	0
**LBD (SNCA Dup)**	63	M	3	1	2
**LBD (SNCA Tri)**	48	M	4	1	0

Table S7. Patient characteristics of the postmortem human brains for qPCR validation

	Control (N=11)	LBD (N=11)
**Age (years)**	77.4 (49.0, 97.0)	70.5 (55.0, 83.0)
**Sex (Male)**	8 (72.7%)	10 (90.9%)
**Thal phase**		
**0**	4 (18.2%)	5 (36.4%)
**1**	2 (27.3%)	6 (18.2%)
**2**	4 (45.5%)	0 (36.4%)
**3**	1 (9.1%)	0 (9.1%)
**Braak stage**		
**0**	2 (18.2%)	2 (18.2%)
**I**	3 (27.3%)	2 (18.2%)
**II**	5 (45.5%)	4 (36.4%)
**III**	1 (9.1%)	3 (27.3%)

The sample median (minimum, maximum) is given for age.

Table S8. Patient characteristics of the postmortem human brains for western blotting validation

	Control (N=16)	LBD (N=16)
**Age (years)**	83.8 (79.0, 91.0)	72.9 (61.0, 83.0)
**Sex (Male)**	9 (56.3%)	14 (87.5%)
**Thal phase**		
**0**	5 (31.3%)	7 (43.8%)
**1**	2 (12.5%)	9 (56.3%)
**2**	5 (31.3%)	0 (0.0%)
**3**	4 (25.0%)	0 (0.0%)
**Braak stage**		
**0**	3 (18.8%)	4 (25.0%)
**I**	3 (18.8%)	3 (18.8%)
**II**	7 (43.8%)	6 (37.5%)
**III**	3 (18.8%)	3 (18.8%)

The sample median (minimum, maximum) is given for age.

Auxiliary Supplementary Materials

Table S2. The list of DEGs from organoid scRNA-seq

Full list of DEGs in each cluster from organoid scRNA-seq. Immature excitatory neuron (ImEX); Immature inhibitory neuron (ImIN); Excitatory neuron 1 (EX1); Excitatory neuron 2 (EX2); Excitatory neuron 3 (EX3); Inhibitory neuron (IN); Radial glia progenitor (RGP); Radial glia 1 (RG1); Radial glia 2 (RG2); Astrocyte (AS). The table is provided as a separate file.

Table S3. The list of DEG-enriched pathways from organoid scRNA-seq

Full list of DEG-enriched pathways in each cluster from organoid scRNA-seq. Immature excitatory neuron (ImEX); Immature inhibitory neuron (ImIN); Excitatory neuron 1 (EX1); Excitatory neuron 2 (EX2); Inhibitory neuron (IN); Radial glia progenitor (RGP); Radial glia 2 (RG2); Astrocyte (AS). The table is provided as a separate file.

Table S5. The list of DEGs from human brain snRNA-seq

Full list of DEGs in each cluster from human snRNA-seq. Excitatory neuron (EX); Inhibitory neuron (IN); Oligodendrocyte (OLG); Oligodendrocyte progenitor cell (OPC); Astrocyte (AS); Microglia (MG); Endothelial cell (Endo). The table is provided as a separate file.

Table S6. The list of DEG-enriched pathways from human brain snRNA-seq

Full list of DEG-enriched pathways in each cluster from human snRNA-seq. Excitatory neuron (EX); Inhibitory neuron (IN); Oligodendrocyte (OLG); Oligodendrocyte progenitor cell (OPC); Astrocyte (AS); Microglia (MG). The table is provided as a separate file.

Table S9. The RT-QuIC signal with drug candidate treatment at 5uM, 1uM, 0.5uM concentration

The values in the aggregation curve of α-SYN seeding activity with the final 8 selected drugs (D1-D8) at different concentrations. Negative (DMSO) and positive (EGCG) controls were included. Entacapone (D1); Carbidopa (D2); Tolcapone (D3); Phenazopyridine hydrochloride (D4); Dyclonine hydrochloride (D5);

Zalcitabine (D6); Methyldopate hydrochloride (D7); Racepinephrine hydrochloride (D8). The table is provided as a separate file.

Table S10. The list of organoid scRNA-seq sample index

The sample characteristics of organoid scRNA-seq samples including sample name, genotype, line ID and colony ID, with indication of an excluded sample. The table is provided as a separate file.
